# Phylotranscriptomics and genome-size evidence clarify the Taiwanese *Cirsium japonicum* complex and delimit *C. brevicaule* and allied East Asian thistles

**DOI:** 10.1186/s12870-026-08097-6

**Published:** 2026-02-05

**Authors:** Chih-Yi Chang, Pei-Chun Liao, Hsy-Yu Tzeng, Junko Kusumi, Zhi-Hui Su, Yen-Hsueh Tseng

**Affiliations:** 1 Zhongzheng Dist., Taiwan Forestry Research Institute, No. 53, Nanhai Rd., Taipei City, 10066 Taiwan; 2 National Museum of Natural Science, Biology Department, Vascular Plants Division, No. 1, Guanqian Rd., North District, Taichung , 40453 Taiwan; 3 School of Life Science, National Taiwan Normal University, No. 88, Sec. 4, Ting-Chow Rd., Wenshan Dist. 116, Taipei City, Taiwan; 4 Department of Forestry, National Chung Hsing University, No. 145, Hsing Ta Rd, 402 Taichung City, Taiwan; 5 Department of Environmental Changes, Faculty of Social and Cultural Studies, Kyushu University, Fukuoka, 819-0395 Japan; 6 JT Biohistory Research Hall, Takatsuki, Osaka 569-1125 Japan; 7 Department of Biological Sciences, Graduate School of Science, Osaka University, Osaka, 560-0043 Japan

**Keywords:** Phylotranscriptomics, Genome-size variation, Island biogeography, Quaternary glaciations, Coalescent inference, Paleodemography

## Abstract

**Background:**

Rapid diversification in island floras often creates taxonomic uncertainty, particularly for morphologically variable plant complexes. The *Cirsium japonicum* complex, a widespread and medicinally important group in East Asia, exemplifies this challenge, with unclear species boundaries and conflicting varietal definitions. This is especially true in Taiwan, where multiple endemic forms co-occur. Despite its ecological and pharmacological significance, due to morphological overlap, fragmented distributions, and the absence of comprehensive phylogenomic analysis, the evolutionary relationships and lineage delimitations within this complex remain unresolved. In this study, we analyzed phylotranscriptomic data from 37 thistle accessions comprising the *C. japonicum* complex and the allied *C. brevicaule* group, constructed multigene coalescent species trees, and integrated morphometrics, genome size mapping, demographic history, and distribution modeling.

**Results:**

Three monophyletic subsections (*Sinocirsium*, *Arenicola*, and *Nipponocirsium*; all within *Cirsium* sect*. Onotrophe*) diverged 1.30–1.02 million years ago (Mya) (95% highest posterior density (HPD): 1.62–0.71 Mya) during early Quaternary glaciations. Within subsect. *Sinocirsium*, five lineages emerged: Japanese var. *japonicum* and four Taiwanese varieties that form two sister pairs (var. *albescens* vs. var. *takaoense* and var. *australe* vs. var. *fukienense*). In subsect. *Arenicola*, two distinct species endemic to the Ryukyu Islands, *C. brevicaule* and *C. irumtiense*, exist across the Miyako Strait. The ancestral genome size was estimated at ~1.3 pg and underwent several independent reductions (e.g., var. *takaoense*, 1.01 pg) and expansions (e.g., *C. brevicaule*, 1.93 pg) without chromosomal changes. Skyline plots indicate a late Pleistocene bottleneck and Holocene rebound in var. *takaoense*, whereas var. *fukienense* remained stable, matching historical habitat suitability.

**Conclusions:**

We combined phylotranscriptomic trees, genome-size trajectories, and demographic models to resolve East Asian *Cirsium* into two Ryukyu species (*C. brevicaule*, *C. irumtiense*) and a five-lineage *C. japonicum* complex spanning Taiwan to Japan. Revised diagnoses confirm that true *C. brevicaule* is confined to the central Ryukyus, while Taiwanese records reflect the complex, including var. *takaoense*, *australe*, *fukienense*, and the Hengchun endemic var. *albescens*, which meets IUCN vulnerability criteria due to restricted range and medicinal harvest. We further clarify the placement of *C. morii*, link floral-color polymorphism in var. *takaoense* to anthocyanin expression and pollinator preference, and show that Quaternary glaciations, island fragmentation, and 2C shifts shaped diversification, whereas late Pleistocene bottlenecks and Holocene expansions structured populations. Together, this synthesis refines taxonomy, informs conservation, and supports sustainable use.

**Supplementary Information:**

The online version contains supplementary material available at 10.1186/s12870-026-08097-6.

## Background

Species differentiation often reflects geographic and historical factors [1–3]. Taiwan experienced major sea-level fluctuations during recent glacial periods, with low sea levels forming land bridges, enabling species migration from surrounding regions. As sea levels rose post-glaciation, these bridges disappeared, isolating populations from mainland counterparts [4–7]. Overall, this warming likely facilitated adaptive radiation and species diversification [8–10].

The genus *Cirsium* Mill. (Compositae) comprises biennial or perennial herbaceous spiny plants characterized by imbricate phyllaries that are often spinose-tipped, densely bristly receptacles, homogamous tubular florets, and cypselae bearing a plumose pappus in many series [11]. Approximately 250–300 species occur across Europe, Asia, North Africa, North America, and Central America [12–14], although recent works have proposed segregating certain lineages from *Cirsium* (e.g., [15–18]; see also [19]), including lineages corresponding to genera such as *Lophiolepis* (Cass.) Cass., *Afrocirsium* Calleja, N. Garcia, Moreyra & Susanna, and *Ascalea* Hill. *Cirsium* diversity is especially notable in the temperate Northern Hemisphere, reflecting marked ecological adaptability [20–22]. Thus, *Cirsium* is an ideal group for studying species diversification.

The early infrageneric framework of *Cirsium* was outlined by de Candolle & Duby [23], who recognized four groups largely based on European species. Subsequently, several authors expanded or modified these early concepts, and de Candolle later synthesized these developments in his 1837 treatment [24], consolidating the genus into six sections (e.g., *Cirsium* sect. *Eriolepis* (Cass.) DC., sect. *Onotrophe* (Cass.) DC., sect. *Lophiolepis* (Cass.) DC., sect. *Orthocentron* (Cass.) DC., sect. *Cephalonoplos* (Neck.) DC., sect. *Corynotrichum* DC.). Because these systems were derived primarily from European species, they were not applicable to East Asian lineages [25]. Kitamura [25] therefore reinterpreted de Candolle’s concepts for the flora of Japan, Korea, and Taiwan, dividing the regional species into three sections.

Taiwan’s geography strongly influences the evolutionary history of native *Cirsium* species [26–30]. Most alpine *Cirsium* species in Taiwan are endemic, whereas the lowland species show close affinities to taxa occurring in neighboring regions [11, 28]. Other species, such as *C. brevicaule* A.Gray and related taxa, present particular taxonomic challenges [12, 25, 31, 32]. *Cirsium albescens* Kitam., first described from Eluanbi Cape in southern Taiwan [33], was later synonymized under *C. brevicaule* [31]. The type specimen of *C. brevicaule* originates from Okinawa, and occurs along the Ryukyu Archipelago [32]. Likewise, *C. irumtiense* Kitam., described from Iriomote Island [26], was subsequently treated as *C. brevicaule* A.Gray var. *irumtiense* (Kitam.) Kitam [25]. and later synonymized under *C. brevicaule* [12]. These taxa belong to *Cirsium* sect. *Onotrophe* subsect. *Arenicola* Kitam. [12, 25, 34], whose members bear broad involucral phyllaries and are confined to the coast [25]. In Taiwan, this subsection also includes *C. morii* Hayata [25].

Consistent morphological differences exist between *C. brevicaule* and *C. irumtiense*, including flower color (*C. brevicaule* white vs. *C. irumtiense* bluish-purple), leaf pubescence (*C. brevicaule* with less pubescence), and plant size (*C. irumtiense* larger). Populations in Taiwan identified as *C. albescens* also differ from type-locality populations of *C. brevicaule* and *C. irumtiense*, having smaller size, extensively branched stems, and narrower involucral phyllaries [11]. Overall, Taiwanese plants appear morphologically closer to the *C. japonicum* DC. species complex.

Resolving classification issues requires understanding the underlying phylogeny; however, relevant studies in East Asia remain scarce. Historically, infraspecific taxa within *C. japonicum* (*Cirsium* sect. *Onotrophe* subsect. *Sinocirsium* Kitam.) have been recognized [25, 35–37]. Subsection *Sinocirsium* is characterized by narrow phyllaries [25]. Another view [38] interprets variation in vegetative organs as related to habitat, leading to the treatment of these taxa as a single species, a classification adopted by both *Flora Reipublicae Popularis Sinicae* [14] and *Flora of China* [39]. However, reproductive characters provide the most reliable distinctions among taxa. For example, *C. japonicum* var. *australe* differs from var. *japonicum* by its longer, narrower corolla tube [40, 41]. Moreover, many studies still support recognizing subdivisions within *C. japonicum* in East Asia [11, 12, 31, 40, 42, 43].

In Taiwan, classification within the *C. japonicum* complex has often relied on flower color. Specifically, white-flowered plants have traditionally been assigned to *C. japonicum* DC. var. *takaoense* Kitam. and bluish-purple-flowered plants to var. *australe* Kitam. [11, 29, 31]. Whether this variation has evolutionary significance remains unclear. Recent surveys suggest that southern Taiwanese populations with bluish-purple flowers resemble var. *takaoense* morphologically, with larger leaves, shallower lobes, and longer internodes, complicating this distinction. Furthermore, *C. japonicum* DC. var. *fukienense* Kitam shows marked flower color variation [29], adding further complexity.

Although *Cirsium* phylogeny has been studied, East Asian taxa remain underrepresented. Few species have been included, with *C. lineare* (*Cirsium* sect. *Spanioptilon*) placed in a basal clade, and *C. japonicum* (subsect. *Sinocirsium*) and *C. nipponicum* (*Cirsium* sect. *Onotrophe* subsect. *Nipponocirsium* Kitam.) in more derived clades [19, 21, 44]. A recent phylogenomic study with broad sampling across Eurasia, East Asia, and North America [45] improved the resolution of these relationships, supporting the affinity between *C. japonicum* and *C. nipponicum* together with multiple species of subsect. *Nipponocirsium*, and confirming the distant placement of *C. lineare*. This expanded dataset also addresses earlier limitations in North American sampling, which previously relied on a small number of markers [21, 22, 46, 47]. Nevertheless, Taiwanese and Ryukyu taxa remain underrepresented in these large-scale studies, and their relationships require further investigation. Our recent transcriptomic analysis of subsect. *Nipponocirsium* yielded high-resolution results [28], demonstrating that transcriptomic data facilitate phylogenetic inference, particularly during recent speciation.

However, the evolutionary relationships within the *C. japonicum* complex remain unclear. Key questions include whether morphological differences are sufficient to define evolutionary lineages, and whether lineages represent incipient species. We hypothesize that (1) Taiwanese varieties of *C. japonicum* form distinct monophyletic lineages congruent with morphological and genome size differences; (2) *C. brevicaule* and *C. irumtiense* are distinct species separated by geographic barriers and genomic traits; and (3) demographic histories can be inferred from genomic data and reflect different responses to late Quaternary climate change.

To test these hypotheses, we reconstructed phylogenetic relationships using transcriptomic data. We also incorporate members of subsect. *Nipponocirsium* [28]. Using morphological and flow cytometry data, we analyzed the demographic histories of two widespread Taiwanese varieties (var. *fukienense* and var. *takaoense*) using extended Bayesian skyline plots (EBSPs) and species distribution modeling (SDM) to infer population dynamic changes. Since the *C. japonicum* complex and *C. brevicaule* are traditional medicinal plants [48–54], taxonomic misidentification may impede their use and hinder conservation, making further clarification necessary.

## Methods

### RNA sequencing

We sampled four varieties of the *C. japonicum* species complex (subsect. *Sinocirsium*) from Taiwan and its outlying islands: var. *albescens*, var. *australe*, var. *fukienense*, and var. *takaoense*, and the nominal var. *japonicum* (from Kyushu, Japan). We included three taxa from subsect. *Arenicola* (*C. morii*, *C. brevicaule*, and *C. irumtiense*) from Taiwan and the Ryukyus [25]. Each taxon comprised 2–6 individuals, totaling 25 samples. We also incorporated three species from subsect. *Nipponocirsium* and *C. lineare* (Thunb.) Sch.Bip. [28], each with two individuals. This yielded 12 taxa and 33 *Cirsium* samples. Our sampling encompasses all Taiwanese members of the *C. japonicum* complex and includes representative Japanese and Ryukyu lineages that bracket the focal group. This coverage provides the taxonomic context needed to resolve relationships and divergence within the complex. To refine calibration points for divergence-time estimation, we added transcriptome data for four non-*Cirsium* species retrieved from the National Center for Biotechnology Information (NCBI). In total, 16 taxa and 37 samples were analyzed (Supplementary Table S1).

Fresh leaves were collected and preserved in RNAlater (Bioman Scientific, New Taipei City, Taiwan; catalog no. TRP010.500) at − 20 °C. Total RNA was then isolated via a modified CTAB protocol [55–58] in which polyvinylpyrrolidone (PVPP) and high-concentration sodium chloride were added to reduce polyphenol and polysaccharide contamination [56, 58]. Total RNA (1 µg per sample) was used for library construction with the *TruSeq Stranded mRNA Library Prep Kit* (Illumina, San Diego, CA, USA) following the manufacturer’s protocol. Briefly, poly(A) + mRNA was isolated using oligo(dT)-coupled magnetic beads and fragmented under elevated temperature. First-strand cDNA was synthesized using random primers, followed by second-strand synthesis, end repair, 3′-adenylation, and adaptor ligation. Libraries were enriched by PCR and purified using the AMPure XP system (Beckman Coulter, Beverly, USA). Library quality was assessed on a Qsep400 System (BiOptic Inc., Taiwan) and quantified using a Qubit 2.0 Fluorometer (Thermo Scientific, Waltham, MA, USA). Sequencing was performed on an Illumina NovaSeq platform to generate 150-bp paired-end reads (Genomics, BioSci & Tech Co., New Taipei City, Taiwan). Raw reads were assessed for quality using FastQC v0.11.9 [59] and summarized with MultiQC v1.12 [60]. Adapter removal and trimming were performed with Trimmomatic v0.39 [61] in paired-end mode. Reads were trimmed using the following settings: ILLUMINACLIP:TruSeq3-PE.fa:2:30:10:8:true, LEADING:3, TRAILING:3, SLIDINGWINDOW:4:15, and MINLEN:100. The resulting paired-end reads were retained for all downstream analyses.

All raw reads have been deposited in the NCBI Sequence Read Archive under BioProject accession PRJNA1311153. All plant materials used for RNA sequencing were formally identified by Chih-Yi Chang. Representative voucher specimens corresponding to these sequenced individuals have been deposited in the Herbarium of the Department of Botany, National Museum of Natural Science, Taichung, Taiwan (TNM), and in the Herbarium of the Department of Forestry, National Chung Hsing University, Taichung, Taiwan (TCF). Voucher accession numbers are listed in Supplementary Table S1.

### De novo assembly and orthologous gene identification

We performed de novo genome assembly as per the protocol specified by Freedman and Weeks [62]. Ribosomal RNA (rRNA) was removed with Bowtie 2 v2.4.2 [63] by aligning reads to sequences found in the Silva rRNA database [64]. Overrepresented sequences were eliminated with the previously published script “RemoveFastqcOverrepSequenceReads.py” [62]. The resulting reads were assembled in Trinity v2.12.0 [65]. Duplicate genes were excluded with CD-HIT-EST v4.8.1 [66, 67] at a threshold of 0.85 identity. Assembly quality was assessed with BUSCO v5.3.2 [68] against the Embryophyta odb9 database, and coding sequences were predicted with TransDecoder v5.5.0 [69]. Orthologous genes (OGs) and gene copy numbers were determined with OrthoFinder v2.5.4 [70]. Each gene cluster was aligned with MACSE v2.06 [71], and ambiguous sites were trimmed in trimAl v1.4.1 [72] using the following parameters: -gt 0.2 -seqoverlap 80 -resoverlap 0.8.

### Phylogeny reconstruction

We reconstructed phylogenetic relationships using both a multispecies coalescent framework and a concatenated approach. Single-copy OGs shared by at least 80% of the samples were selected for phylogenetic and split tree analyses, with all samples included in the Bayesian procedure. Individual gene trees were inferred in RAxML v8 [73] under the GTRCAT model with 100 bootstrap replicates, then combined in ASTRAL v5.7.7 [74] using default settings. The resulting all-sample species tree was rooted at *G. ventosus* and at *C. lineare* for the *Cirsium* subset. Visualization used FigTree v1.4.3 [75].

To reduce the computational load of BEAST v2.6.3 [76, 77], we analyzed 50 single-copy orthogroups retained after filtering by taxon coverage, alignment quality, and AMAS-based [78] polymorphism statistics, using StarBEAST3 [79] as implemented in BEAUti v2.6.3. Each gene used a strict clock, a ploidy value of 2.0, and a site model chosen by jModelTest v2.1.10 [80, 81], following Bagley [82]. We ran 100 million generations, sampling every 1,000, under a Yule model with default priors (uniform distribution [0, ∞]). Convergence (ESS > 200) was confirmed in Tracer v1.7.1 [83]. Topologies were visualized with DensiTree v2.2.7 [84], and the maximum clade credibility tree was summarized in TreeAnnotator v2.6.3 [85] with a 10% burn-in before final editing in FigTree v1.4.3. For the concatenated analysis, aligned gene sequences were merged into a supermatrix with FasParser v2.13.0 [86]. Neighbor-net networks were inferred using the SplitsTree4 algorithm implemented in the SplitsTree App [87]. For this analysis, we excluded *C. lineare* to mitigate long-branch attraction.

### Species delimitation

To minimize outgroup effects, delimitation was restricted to *Cirsium* taxa. Species boundaries were tested using discovery methods based on individual gene trees [88]. A rooted ASTRAL species tree was analyzed in SODA v1.0.2 [89] with thresholds of 0.005, 0.01, and 0.05. Multirate Poisson tree processes (mPTPs) and MCMC-mPTPs were implemented on the mPTP web servers [90] under default settings. The generalized mixed Yule coalescent (GMYC) method [90, 91] was also applied to a Bayesian species tree, using both single- and multithreshold GMYC approaches under default parameters.

### Divergence time estimation

BEAST settings followed those used for our phylogenetic analyses. To refine divergence estimates, additional non-*Cirsium* taxa were included (Supplementary Table S1) and two calibration points (CPs) were applied. These included CP1, set at 83.5 Mya, representing the origin of the Compositae [19, 92], and CP2, fixed at 14 Mya, based on *Cirsium* achene fossils [93]. CP2 was placed between the *Cynara* and *Silybum* nodes [19]. Both followed normal distributions with a standard deviation of 0.5. Major Quaternary glacial events [94] were marked on the resulting timeline.

### Genome size measurement and ancestral state reconstruction

To assess genome size variation within the *C. japonicum* complex (subsect. *Sinocirsium*) and allied taxa from subsect. *Arenicola*, we measured nuclear DNA content (2C values) of 25 accessions (≥ 3 per taxon) [95–97]. Young leaves were ground in 500 µL of CyStain™ PI Absolute P nuclei-extraction buffer (Sysmex Partec, Görlitz, Germany) together with the standard *Solanum lycopersicum* (2C = 2.0 pg [98, 99]). Samples were then filtered through a 40 µm mesh, stained in 2 mL of buffer containing 12 µL of propidium iodide and 6 µL of RNase A, incubated on ice for 30 min, and analyzed on a BD FACSAria III flow cytometer (488 nm, 15 mW). Propidium-iodide fluorescence (620/645 nm) from ≥ 5,000 nuclei per sample was recorded and processed with FlowJo v10.10. Genome sizes were then calculated from sample-to-standard fluorescence ratios (1 pg ≈ 978 Mb).

A species tree was then reconstructed with ASTRAL v5.7.7 [74] under the above parameters, using one voucher specimen per taxon (Supplementary Table S3). Ancestral genome-size states were inferred with the fastAnc() function in the R package *phytools* v2.2–0 [100, 101]. Plant individuals subjected to genome size measurement were also formally identified by Chih-Yi Chang. Voucher specimens for these cytometric samples have been deposited in TNM and TCF. The corresponding voucher numbers are provided in Supplementary Table S4.

### Extended Bayesian skyline plot (EBSP)

To elucidate population dynamics within the Taiwanese *C. japonicum* complex, we conducted EBSP analyses for two widely distributed but nonoverlapping varieties: var. *fukienense* (four populations) and var. *takaoense* (six populations). From the pool of OGs (orthologous groups) shared by all OTUs (operational taxon units), we selected 56 OGs for var. *fukienense* and 60 OGs for var. *takaoense*, each set including loci with the highest SNP counts. We applied the StarBEAST module [76] implemented in BEAUti v2.6.3 [77] and performed BLASTn searches (NCBI remote mode) against the nt database [102] to determine gene origins. Uncertain matches or those lacking hits were assumed to be nuclear genes. BEAST settings largely followed earlier phylogenetic analyses, following the EBSP approach [103]. A strict clock was used, with evolutionary rates set to 0.05 for mitochondrial and chloroplast genes and 0.005 for nuclear genes, allowing estimation in the program. A coalescent extended Bayesian skyline model [103] was used for species tree evolution. MCMC chains ran for 10,000,000 generations with sampling every 10,000 (treelog). EBSPs were plotted in R v4.3.1 [100] using the plotEBSP.R script [103].

### Species distribution modeling (SDM)

For SDM, we focused on two varieties of the *C. japonicum* complex, var. *fukienense* and var. *takaoense*. Records were compiled from field surveys and herbarium specimens within Taiwan, supplemented with iNaturalist data from Fujian Province, China, since the native range spans both Taiwan and adjacent Fujian coasts. All records were checked to remove misidentified points. To ensure spatial independence, points within 500 m were merged. The final dataset (Supplementary Figure S4) comprised 56 points for var. *fukienense* and 51 for var. *takaoense*.

Following a previous approach [104] with minor modifications, we obtained 19 bioclimatic variables (1970–2000) from WorldClim v2.1 [105] at ~ 1 km resolution, cropped to the Taiwan area and the adjacent Fujian coast (118°E–123°E, 21.5°N–26.5°N, WGS84/EPSG:4326). Using the R *raster* package [106], we calculated Pearson’s correlation coefficients and excluded all with |r|> 0.8. This yielded seven variables: mean diurnal range (BIO2), isothermality (BIO3), mean temperature of the driest (BIO9) and warmest (BIO10) quarters, precipitation seasonality (BIO15), and precipitation of the warmest (BIO18) and coldest (BIO19) quarters.

SDMs were generated with Maxent v3.4.4 [107], with each run including 1,000 iterations, 10,000 background points, and a regularization multiplier of one. Ten bootstrap replicates were performed per model, and performance was evaluated by the area under the ROC curve (AUC), with 20% of occurrence points randomly sampled and set aside for testing.

For past distributions, SDMs were projected onto paleoclimate layers from CHELSA TraCE21k [108] using the seven bioclimatic variables. Sea level corrections were applied for each period [109–111]: + 2.5 m for 3 kya, − 20 m for 9 kya, − 50 m for 12 kya, and − 120 m for 20 kya and the Last Glacial Maximum (~ 21 kya). Digital elevation model (DEM) data for Taiwan and adjacent coasts were obtained from geodata [112], mosaicked, and cropped. DEMs represented terrestrial elevations, while NOAA’s ETOPO1 global bathymetry dataset [113, 114] was used where DEM data were unavailable, mainly in offshore areas. Land masks were generated by applying sea level thresholds and retaining only cells above adjusted levels.

Land masks were resampled to match the bioclimatic layers, then applied to remove submerged areas before SDM projection. We projected the predicted distributions of the two varieties onto the resulting maps.

### Morphological comparisons

To clarify taxonomic relationships, we compared morphological traits across two assemblages, each containing three OTUs. There were the *C. brevicaule* group, comprising representative populations of *C. brevicaule*, *C. irumtiense* (≡ *C. brevicaule* var. *irumtiense*), and *C. japonicum* var. *albescens* (≡ *C. albescens*; = *C. brevicaule*); and the *C. japonicum* complex, consisting of white- and bluish-purple-flowered populations of var. *takaoense* and populations of var. *australe*.

Morphological variation was also examined in fresh and herbarium specimens. Voucher samples were deposited in the Herbarium, Department of Forestry, National Chung Hsing University (TCF), and the Herbarium of the National Museum of Natural Science, Taichung (TNM). Additional material was examined from CHIA, HAST, PPI, TAI, TAIE, TAIF, TCF, TI, TNM, and TNU. We also reviewed high-resolution images and metadata from the AU, KUN, PE, IBSC, HHBG, NAS, and TNS online databases. Herbarium acronyms follow the Index Herbariorum [115].

For each OTU, morphological measurements were obtained from more than three populations, and each character was measured from at least three individuals per population. Morphological terminology follows [11, 44]. Quantitative data are presented as mean ± standard deviations. Taxonomic differences were tested by one-way ANOVA followed by Tukey’s HSD multiple-range test [116]. Statistical analyses were performed in PASW Statistics v18 [117].

## Results

### Bioinformatics analyses

Raw sequencing data from the 25 samples generated 1,252,725,046 reads, averaging 50,109,002 reads per sample, with a mean GC content of 47%. For the additional 12 samples obtained from NCBI (4 samples) and other studies (8 samples) [28], we obtained 52,417,004 reads, averaging 46,034,750 reads per sample and a mean GC content of 46%. In total, we obtained 37 samples yielding 1,805,142,050 reads, with an average of 48,787,623 reads per sample and an overall mean GC content of 47%.

After trimming, rRNA removal, and elimination of overrepresented sequences, approximately 676.9 million high-quality reads remained, averaging 18.29 million reads per sample, with a mean GC content of 45%. The initial average proportion of complete BUSCOs was 72.16%, with duplicate genes comprising 28.93%. Following duplicate removal, the average proportion of complete BUSCOs slightly declined to 71.87%, while the proportion of duplicate genes dropped markedly to 10.65% (Supplementary Table S1).

A total of 59,186 OGs were identified from the 37 samples, of which 3,157 single-copy OGs shared by at least 80% of the samples were selected. After alignment and trimming of ambiguous sites, 2,999 OGs were retained for phylogenetic analyses via ASTRAL and SplitsTree, totaling 2,880,714 bp and containing 667,007 SNPs. These OGs averaged 961 bp in length and 222 SNPs each. For BEAST analysis, 52 OGs shared by all samples were retained, totaling 34,533 bp with 9,375 SNPs, and averaging 664 bp and 180 SNPs per OG.

From the 33 *Cirsium* samples used for species delimitation, 57,984 OGs were identified. Of these, 2,599 single-copy OGs shared by at least 85% of the samples were selected. After alignment and trimming, 2,442 OGs were retained for ASTRAL analysis, totaling 2,136,612 bp with 116,283 SNPs, averaging 875 bp and 48 SNPs each. For BEAST analysis, 53 OGs shared by all samples were retained, totaling 32,856 bp and 1,310 SNPs, with a mean length of 620 bp and 25 SNPs each.

### Phylotranscriptomic insights

**Fig. 1:**
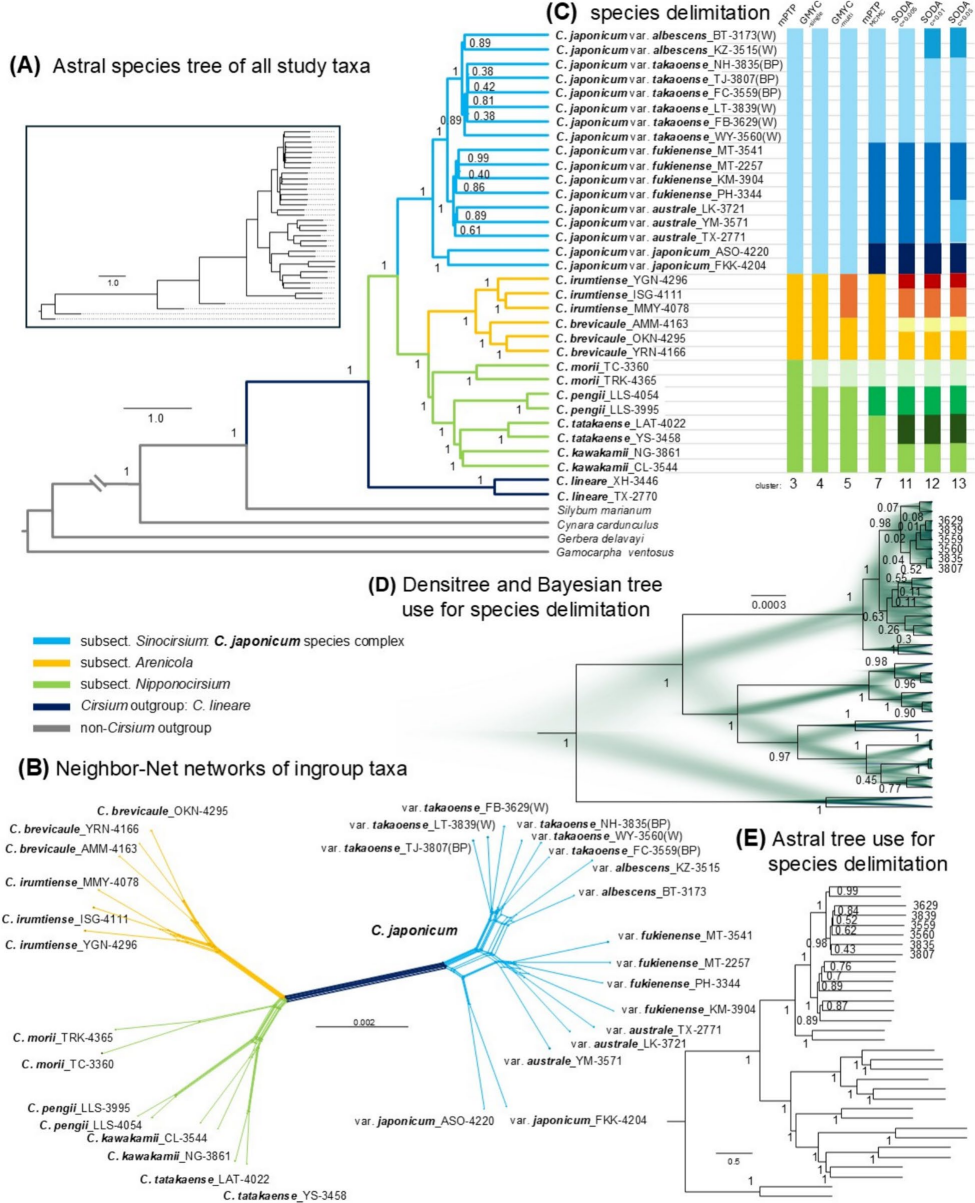
Phylotranscriptomic reconstruction and species delimitation of *Cirsium* subsections *Nipponocirsium*, *Arenicola*, and *Sinocirsium* (i.e., the *C. japonicum* complex). **A** ASTRAL species tree of all sampled taxa based on 2,999 orthologous gene (OG) trees. The whole tree topology is shown in the inset. **B** Neighbor-Net network of ingroup taxa inferred from the concatenated single-copy sequences of 2,599 OGs. **C** Species delimitation results. GMYC was applied to the Bayesian tree based on information from 52 OGs (panel **D**), whereas SODA and PTP analyses were conducted using the ASTRAL species tree inferred from 2,599 OGs (panel **E**). Different colors represent clusters identified using different methods. **D** *Densi tree* visualization and consensus tree of *Cirsium* taxa based on the posterior distributions of Bayesian trees inferred from 52 OGs. **E** ASTRAL species tree used for SODA and PTP species delimitation analyses. Node numerical values shown in panels **A**, **D**, and **E** represent posterior probabilities (PPs). Tip label numbers in panels D and E indicate samples showing topological conflict relative to the ASTRAL species tree depicted in panel **A**. For *C. japonicum* var. *takaoense*, (W) denotes the white-corolla morph, and (BP) denotes the bluish-purple-corolla morph

The species tree based on 2,999 OGs, inferred via ASTRAL (Fig. 1A), revealed well-resolved phylogenetic relationships among *Cirsium* ingroup taxa, with strong local posterior probabilities. Subsections *Arenicola* and *Nipponocirsium* formed a well-supported clade (posterior probability [PP] = 1), resolved as sister to subsect. *Sinocirsium*. All three subsections were monophyletic, indicating deep divergence among major lineages.

Within subsect. *Sinocirsium*, the *C. japonicum* complex was subdivided into three major lineages (PP = 1). Var. *japonicum* was inferred as the basal lineage, while Taiwanese varieties formed two well-supported sister groups: one comprising var. *albescens* and var. *takaoense*, and the other var. *australe* and var. *fukienense*. These four varieties were resolved as reciprocally monophyletic (PP = 1). Internal support was lower within individual varieties, particularly for var. *australe* (PP = 0.61–0.89) and var. *fukienense* (PP = 0.40–0.99). Notably, var. *takaoense* showed low support (PP = 0.38–0.81) and topological variation between the DensiTree, Bayesian tree (Fig. 1D), and the alternative ASTRAL tree used for species delimitation (Fig. 1E). This may reflect recent radiation events.

Subsect. *Arenicola* included the Ryukyu taxa *C. brevicaule* and *C. irumtiense*, while subsect. *Nipponocirsium* included the Taiwanese endemic *C. morii* as the earliest diverging lineage, followed by a clade of *C. pengii*, *C. tatakaense*, and *C. kawakamii*, all strongly supported (PP = 1).

For rooting the ingroup phylogeny, both an intrageneric outgroup (*C. lineare*) and three non-*Cirsium* outgroups (*Cynara cardunculus*, *Gerbera delavayi*, and *Gamocarpha ventosa* (Meyen) S.Denham & Pozner) were used. *Cirsium lineare* provided a close reference without reducing ingroup resolution. Outgroups were clearly separated from the ingroup, supporting the monophyly of the East Asian *Cirsium* lineages.

The neighbor-net network based on 2,599 concatenated single-copy OGs (Fig. 1B) further supported the major clades identified above while revealing reticulate patterns and possible gene flow within the complex. Clear separations were observed among subsections, whereas complex reticulations among *C. japonicum* varieties suggested incomplete lineage sorting or historical introgression. *C. morii* was placed near the base of subsect. *Nipponocirsium* and showed genetic divergence from other core members.

The Bayesian consensus tree and DensiTree visualization (Fig. 1D) closely matched the ASTRAL species tree, with high posterior support and minimal topological conflict along the backbone. The alternative ASTRAL tree used for species delimitation (Fig. 1E) was largely congruent with the main topology, further supporting the phylogenetic framework. Minor discrepancies, as noted above, may reflect recent divergence within Taiwanese members of the *C. japonicum* complex.

### Species delimitation

To assess lineage boundaries and evaluate species hypotheses within East Asian *Cirsium*, we applied three complementary species delimitation approaches (Fig. 1C). The GMYC model was implemented with the Bayesian consensus tree (Fig. 1D), whereas both PTP and SODA were conducted with the ASTRAL species tree (Fig. 1E). Across methods, results were largely consistent, particularly in delimiting taxa corresponding to established subsections. Importantly, all three approaches supported the monophyly of the three major subsections, *Sinocirsium*, *Arenicola*, and *Nipponocirsium*.

To further evaluate boundary robustness, we compared results across seven analytical outputs derived from the three frameworks. Taxa supported by at least four outputs were designated as well delimited. Thus, several lineages, including *C. japonicum* var. *japonicum* within subsect. *Sinocirsium*, as well as *C. morii* and *C. pengii* within subsect. *Nipponocirsium*, showed stable delimitation. In addition, *C. kawakamii* and *C. tatakaense* were consistently delimited under all three SODA criteria, although support from GMYC and PTP was less consistent.

In subsect. *Arenicola*, *C. brevicaule* and *C. irumtiense* were generally supported as distinct units across analyses. Under the GMYC-multi model, they were recovered as two species. However, SODA criteria further subdivided each taxon into two entities, producing four putative lineages. This subdivision may reflect within-species population structure or incipient speciation. In contrast, subsect. *Sinocirsium* showed greater incongruence within the *C. japonicum* complex. *C. japonicum* var. *japonicum* (Japan) and var. *albescens* (Taiwan) were generally recognized as distinct lineages. However, separation of var. *australe* and var. *fukienense* was supported only under the most permissive SODA criterion (c = 0.05). Limited support for their distinction is consistent with their current treatment as intraspecific varieties. These results suggest ongoing diversification and potentially unresolved species boundaries among Taiwanese members of the *C. japonicum* complex.

### Divergence history and Pleistocene radiation

**Fig. 2:**
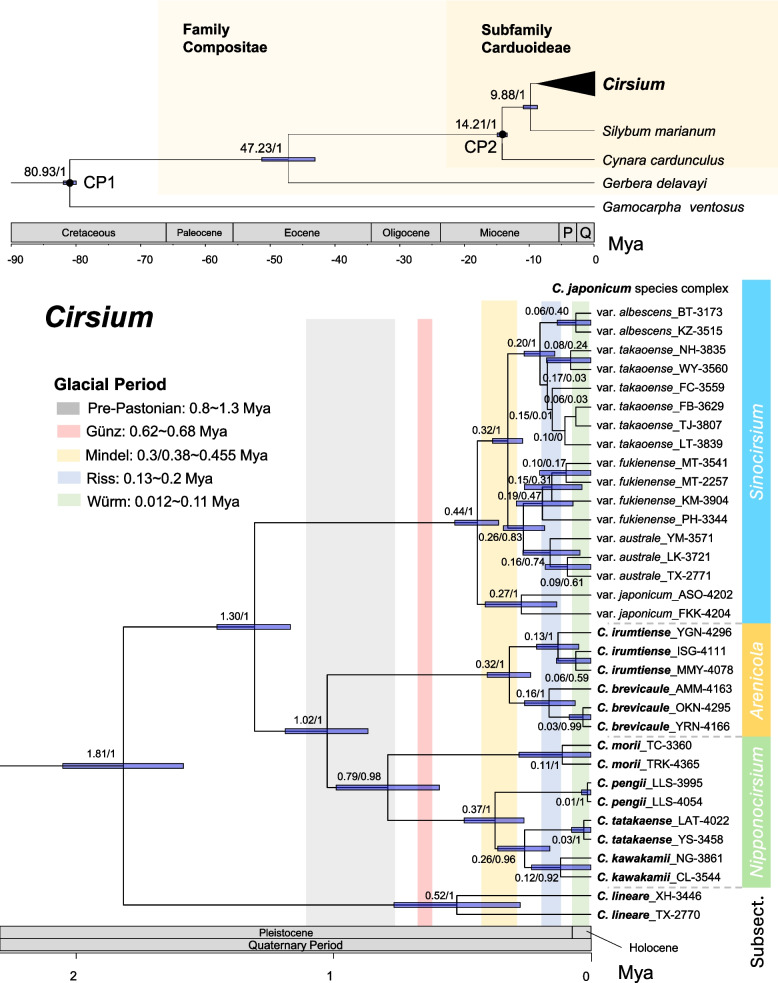
Time-calibrated phylogeny of *Cirsium* subsections *Nipponocirsium*, *Arenicola*, and *Sinocirsium* (i.e., the *C. japonicum* complex) within the Compositae. This tree is inferred from 50 orthologous genes (OGs). Node labels indicate median divergence times and posterior probabilities (PPs). Dark blue bars represent 95% highest posterior density (HPD) intervals for node ages. Black circles mark calibration points (CPs) used for molecular dating. On the time scale, “P” and “Q” refer to Pliocene and Quaternary, respectively. Colored background bands correspond to glacial periods

A time-calibrated phylogeny inferred from 50 OGs (32 nuclear, 17 chloroplast, and 1 mitochondrial) revealed the divergence history of the *Cirsium* subsections, with particular focus on the *C. japonicum* complex (Fig. 2). The divergence-time analysis showed strong overall convergence, with all major parameters (posterior, tree, substitution, clock, priors) having ESS values > 790 (Supplementary Table S2). Although deep-node calibrations in Asteraceae inevitably introduce broad temporal uncertainty, the crown age of *Cirsium* was estimated at ~ 9.88 Mya (95% highest posterior density [HPD]: 7.39–11.74), with strong support at all deep nodes (posterior probability [PP] = 1.0). This estimate falls within the range reported in previous studies, including [92] and [118], which inferred crown ages of ~ 9.1 and ~ 9.7 Mya, respectively. Divergence among the three subsections occurred between 1.30 and 1.02 Mya (95% HPD: 1.62–0.71). This estimate also agrees with the divergence time inferred for the ancestor of *C. lineare* in [45], which was dated to ~ 1.4 Mya (95% CI: 0.7–2.7 Mya), closely overlapping with our estimate of 1.81 Mya (95% HPD: 1.6–2.0 Mya).

Chronological analysis of divergence times reveals how successive isolation events shaped the differentiation of East Asian *Cirsium* lineages. Subsect. *Sinocirsium* diverged from the other two subsections at 1.30 Mya (95% HPD: 1.04–1.62), preceding the pre-Pastonian glacial period. In contrast, the split between subsect. *Nipponocirsium* and subsect. *Arenicola* occurred at 1.02 Mya (95% HPD: 0.71–1.33), roughly corresponding to the pre-Pastonian glaciation. Within subsect. *Nipponocirsium*, *C. morii* diverged from its congeners at ~ 0.79 Mya (95% HPD: 0.43–1.18), spanning the pre-Pastonian to Günz glacial stages. The two species in subsect. *Arenicola*, *C. brevicaule* and *C. irumtiense*, diverged at ~ 0.93 Mya (95% HPD: 0.71–1.33). In subsect. *Sinocirsium*, divergence between the Japanese lineage (*C. japonicum* var. *japonicum*) and the Taiwanese clade was estimated at ~ 0.44 Mya (95% HPD: 0.31–0.66), overlapping with the Günz–Mindel interglacial period and early Mindel glaciation.

The most recent radiation events occurred within the Taiwanese *C. japonicum* complex. Several shallow nodes, including those between var. *albescens* and var. *takaoense* and between var. *fukienense* and var. *australe*, had divergence times ranging from ~ 0.08 to 0.44 Mya (95% HPD). These events coincided with major Pleistocene glacial episodes, including the Mindel, Riss, and Würm glaciations. Overall, this pattern suggests that Pleistocene climatic oscillations may have played a role in driving population fragmentation and lineage diversification within the *C. japonicum* complex.

### Genome size measurement

**Fig. 3:**
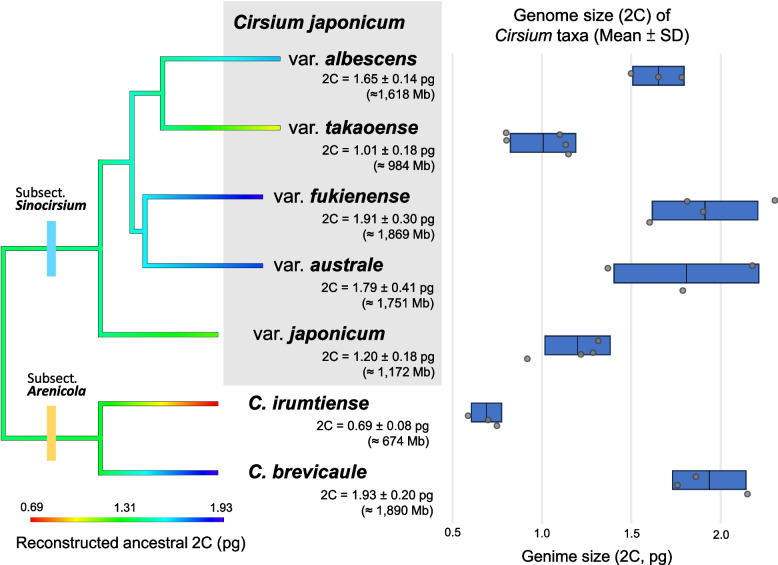
Ancestral reconstruction of genome size and observed 2 C variation among *Cirsium* taxa. Left panel indicates phylogeny with ancestral 2 C reconstruction. Shown is an ASTRAL‐inferred species tree in which each branch is color‐coded according to the reconstructed ancestral 2 C value (in pg). Branch color transitions illustrate inferred increases or decreases in 2 C over evolutionary time. Terminal labels list measured taxon genome size in the format “2C = mean ± SD (pg) (≈ Mb)”. Here the value in megabases (Mb) is calculated assuming 1 pg ≈ 978 Mb. Subsection membership (*Sinocirsium* vs. *Arenicola*) is indicated by colored rectangles at the corresponding nodes. Right panel indicates box‐and‐whisker plots of individual and summary 2 C measurements. For each taxon, a blue horizontal box spans the range of the mean ± 1 SD of 2 C (pg). A vertical line marks the mean. Overlaid gray circles represent individual 2 C measurements for each sample

Next, we constructed an ASTRAL-inferred species tree based on 4,083 OGs (Fig. 3); this generated a topology that was consistent with previous results (Fig. 1). Integration with ancestral 2 C genome size reconstruction suggests that the deepest node of these *Cirsium* lineages had a genome size of ~ 1.3 pg (Fig. 3).

Among the five varieties of *C. japonicum*, var. *takaoense* had the smallest mean genome size (1.01 ± 0.18 pg), whereas var. *fukienense* showed the largest expansion (1.91 ± 0.14 pg). Intermediate values were recorded for var. *japonicum* (1.20 ± 0.11 pg), var. *albescens* (1.65 ± 0.14 pg), and var. *australe* (1.79 ± 0.20 pg). These values indicate a marked reduction in var. *takaoense*, a slight reduction in var. *japonicum*, and moderate expansions in var. *albescens*, var. *australe*, and var. *fukienense* relative to the ancestral estimate (Supplementary Figure S1C–G and Supplementary Table S4 provide individual flow cytometry histograms).

Within subsect. *Arenicola*, *C. irumtiense* exhibited the greatest reduction, with the smallest genome (0.69 ± 0.08 pg), whereas *C. brevicaule* showed the largest genome (1.93 ± 0.20 pg), underscoring lineage-specific trajectories of genome reduction and expansion (Supplementary Figure S1A–B and Supplementary Table S4 provide individual flow cytometry histograms). Since genome-size shifts are scattered throughout the tree, they are best interpreted as independent episodes of expansion or reduction accompanying divergence. Overall, our results indicate that genome size has evolved along multiple, uncoordinated paths.

### Demographic history inferred from the extended Bayesian skyline plot (EBSP) of the two *Cirsium japonicum* varieties

Next, we conducted EBSP analyses on two widely distributed and well-sampled varieties of *C. japonicum* DC, i.e., var. *takaoense* and var. *fukienense*, to investigate historical demographic changes. BLAST searches against GenBank reference sequences were used to assign locus origin for each EBSP dataset. In var. *fukienense*, 56 OGs were retained (45 nuclear, 10 chloroplast, and 1 mitochondrial), whereas var. *takaoense* comprised 60 OGs (46 nuclear, 12 chloroplast, and 2 mitochondrial). Both EBSP analyses showed good convergence, with Ne and skyline parameters all exceeding 320. Only the var. *takaoense* dataset contained 3 of 114 low-information substitution-model parameters with ESS < 200 (90–176), whereas all parameters for var. *fukienense* exceeded 200 (Supplementary Table S2). Histograms of tree event times (Supplementary Figure S2) for both varieties indicated that most coalescent events occurred within the last 0.1 Mya, with very few predating this period. Accordingly, demographic interpretations focus on the last 0.1 Mya, where coalescent support is strongest.

**Fig. 4:**
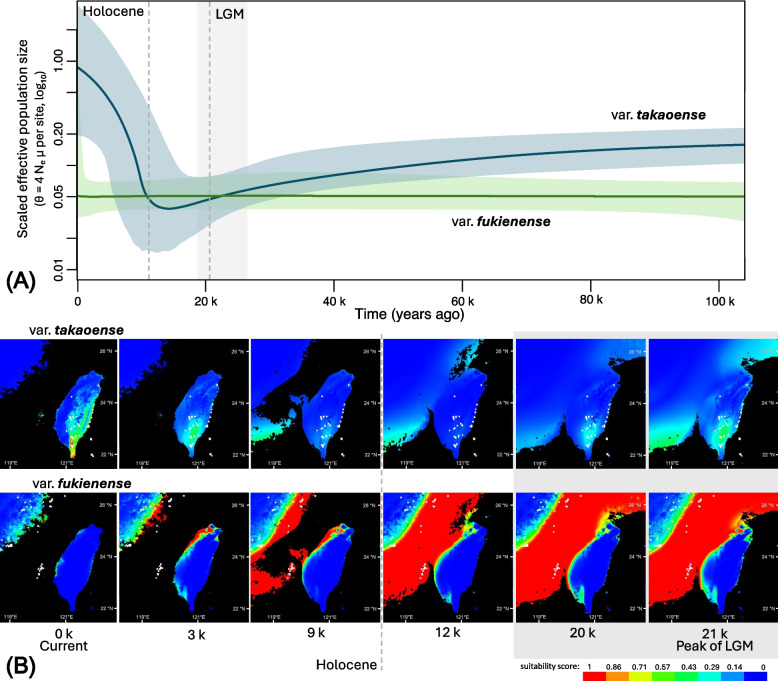
Historical demographic and habitat suitability changes of two varieties of *Cirsium japonicum.***A** Extended Bayesian skyline plot (EBSP) showing historical effective population size changes of *C. japonicum* var. *takaoense* (blue curve) and var. *fukienense* (green curve). Shaded areas represent 95% HPD intervals. Shaded gray area denotes the approximate period of the Last Glacial Maximum (LGM; ~ 26.5–19 thousand years ago [kya]). A dashed line marks the peak of the LGM (~ 21 kya). A second dashed line indicates the onset of the Holocene at approximately 11.7 kya. **B** Species distribution models (SDMs) projected for six paleoclimatic periods (i.e., 0 kya [current], 3 kya, 9 kya, 12 kya, 20 kya, and 21 kya) on the basis of reconstructed climatic conditions. Warmer colors indicate higher habitat suitability scores. White points denote current occurrence records

EBSP analysis (Fig. 4A) revealed markedly different demographic trajectories between var. *takaoense* and var. *fukienense* over the past 0.1 million years. In var. *takaoense*, the scaled effective population size (θ) declined gradually beginning ~ 0.1 Mya, coinciding with the onset of the most recent glacial cycle. This decline continued through the Last Glacial Maximum (LGM; ~ 26.5–19 kya) and reached its lowest point near the onset of the Holocene (~ 11.7 kya). Following the Holocene transition, var. *takaoense* exhibited a pronounced increase in θ, which has persisted to the present. Overall, these results indicate a demographic contraction during the LGM–Holocene interval followed by postglacial expansion.

In contrast, var. *fukienense* maintained a largely stable demographic profile over the same period, with no consistent signal of bottlenecks or expansions associated with the LGM or subsequent climatic fluctuations. Although a modest rise in θ is visible within the 95% HPD intervals, the median trajectory remained stable, suggesting limited demographic perturbation.

These contrasting trajectories suggest that the two varieties responded differently to Quaternary climatic oscillations, potentially reflecting differences in historical habitat stability, ecological breadth, or dispersal capacity.

### Species distribution models (SDMs) of two varieties of *Cirsium japonicum*

SDMs for var. *fukienense* and var. *takaoense* demonstrated strong model performance. The regularized training gain was 2.08 for var. *fukienense* and 1.78 for var. *takaoense*, with high training AUC values of 0.9383 and 0.9403, respectively. Model convergence occurred after 700 iterations for var. *fukienense* and 840 iterations for var. *takaoense*. Projected suitability patterns across paleoclimatic periods revealed contrasting distributional histories (Fig. 4B).

Currently, var. *takaoense* occupies montane and coastal regions of southern and eastern Taiwan. Historically, however, during the LGM (~ 21–20 kya) and before the Holocene, suitable areas contracted, likely reaching a minimum around ~ 12 kya. At that time, suitable habitats were restricted to portions of the exposed continental shelf. Following the onset of the Holocene (~ 11.7 kya) and associated climatic amelioration, suitable areas expanded progressively. By ~ 9 kya, as sea levels rose, suitable habitats shifted toward the Penghu Land Bridge and parts of southern Taiwan. From ~ 3 kya to the present, var. *takaoense* expanded steadily across southern and eastern Taiwan. This spatiotemporal pattern aligns with the demographic contraction and postglacial expansion inferred from EBSP analyses.

In contrast, var. *fukienense* maintained a broader and relatively stable distribution across the continental shelf region throughout the LGM and Holocene. The overall extent of suitable habitat declined gradually with rising sea levels rather than abrupt climatic shifts. At present, var. *fukienense* is restricted mainly to coastal areas of southeastern Fujian, adjacent islands, and the Penghu Archipelago.

In addition to differences in distribution, the two varieties also showed distinct climatic constraints. For var. *takaoense*, the most important environmental predictor was mean diurnal range (BIO2), accounting for 61.70% of the permutation importance. For var. *fukienense*, precipitation in the warmest quarter (i.e., BIO18) was the top variable, contributing 77.13% of the permutation importance. These results suggest that the two varieties are limited by different climatic factors: thermal variability for var. *takaoense* and seasonal precipitation for var. *fukienense*.

### Morphological comparisons

To assess whether morphological divergence corroborates the lineage partitions recovered from molecular analyses, we compared diagnostic traits of six focal OTUs grouped into two independent assemblages.

### Comparative morphology of *Cirsium brevicaule* and allied taxa

**Fig. 5:**
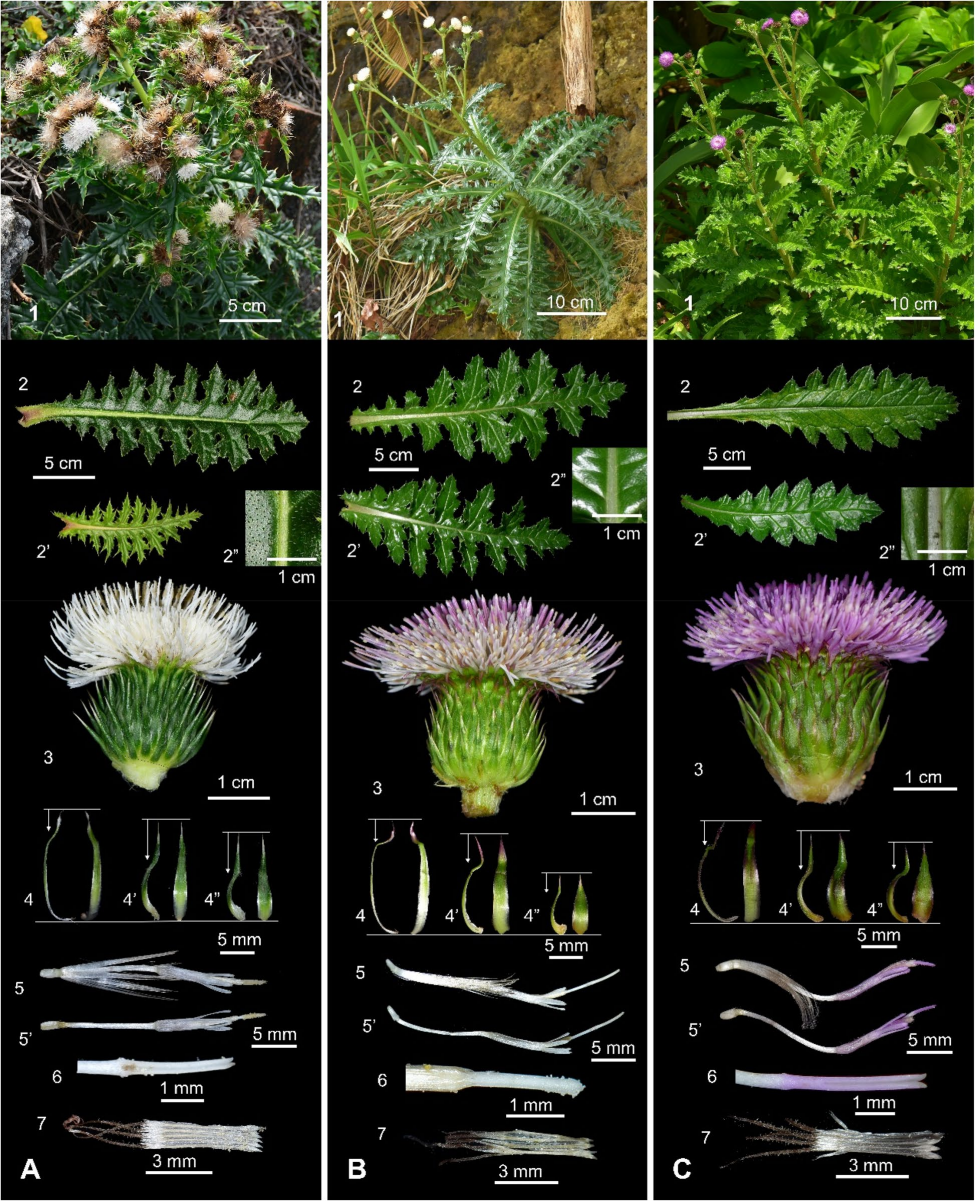
Comparative morphology of *Cirsium brevicaule* A. Gray and allied taxa. **A:** *C. japonicum* DC. var. *albescens* (Kitam.) Y.H. Tseng, P.C. Liao & Chih Y. Chang; **B:** *C. brevicaule* A. Gray; **C:** *C. irumtiense* Kitam. 1: Habit; 2: Rosette leaf; 2’: Cauline leaf; 2″: Adaxial surface 3: Capitula; 4: Inner phyllary; 4’: Middle phyllary; 4″: Outer phyllary; 5: Floret; 5’: Floret (pappus removed); 6: Style branches; 7: Synantherous stamen

Morphological comparisons among *C. japonicum* var. *albescens*, *C. brevicaule*, and *C. irumtiense* revealed several significant differences (Fig. 5, Table 1). Var. *albescens* was significantly smaller in overall plant size: its rosette leaves averaged 12.50 × 4.34 cm, and its cauline leaves averaged 7.89 × 3.36 cm (Fig. 5A1, 2), compared with 32.12 × 10.25 cm and 13.93 × 7.23 cm in *C. brevicaule* (Fig. 5B1, 2) and 31.55 × 11.10 cm and 12.60 × 5.66 cm in *C. irumtiense* (Fig. 5C1, 2). Leaf surfaces of var. *albescens* were hispid (Fig. 5A2″) rather than glabrous or shortly pubescent (Fig. 5B, C2″).

**Table 1:** Comparison of *Cirsium brevicaule* A. Gray and allied taxa

Taxon		*C. japonicum* var. *albescens*	*C. brevicaule*	*C. irumtiense*
Rosette leaves	size (cm)*	12.50 ± 5.34^b^ × 4.34 ± 1.97^b^	32.12 ± 10.36^a^ × 10.25 ± 2.84^a^	31.55 ± 17.91^ab^ × 11.10 ± 5.86^a^
	shape	narrowly elliptic to oblanceolate	narrowly elliptic to oblanceolate	narrowly elliptic to oblanceolate
	adaxial surface	hispid	glabrous to shortly pubescent	shortly pubescent
Cauline leaves	size (cm)*	7.89 ± 2.86^b^ × 3.36 ± 1.05^c^	13.93 ± 3.33^a^ × 7.23 ± 1.04^a^	12.60 ± 3.27^a^ × 5.66 ± 1.14^b^
	shape	narrowly elliptic to narrowly oblong‑elliptic	narrowly elliptic to narrowly oblong‑elliptic	narrowly elliptic to narrowly oblong‑elliptic
	surface	hispid	glabrous to shortly pubescent	shortly pubescent
Capitulum size (cm)*		2.66 ± 0.60^a^ × 1.36 ± 0.32^b^	3.68 ± 0.18^a^ × 1.79 ± 0.14^a^	3.40 ± 0.65^a^ × 1.93 ± 0.36^a^
Phyllary	size (mm)*	17.13 ± 4.38^a^ × 1.72 ± 0.26^b^	10.21 ± 3.50^b^ × 2.58 ± 0.25^a^	13.64 ± 1.56^a^ × 2.76 ± 0.49^a^
	length ratio (inner vs outer)	1.34 ± 0.12^b^	2.24 ± 0.22^a^	1.44 ± 0.07^b^
	protrusion (mm)	5.54 ± 1.4^a^	3.73 ± 0.81^a^	4.44 ± 0.23^a^
Floret	length (cm)	2.36 ± 0.19^c^	2.94 ± 0.08^a^	2.59 ± 0.05^b^
	corolla color	white	white	bluish‑purple
	corolla‑lobe length (mm)	3.47 ± 0.29^a^	3.07 ± 0.27^b^	3.36 ± 0.21^a^
	pappus length (cm)	1.19 ± 0.09^b^	1.41 ± 0.08^a^	1.11 ± 0.02^b^
Distribution		Endemic to Taiwan; currently found only on the Hengchun Peninsula in open areas, grasslands and sandy habitats below 500 m [11, 33]	Endemic to Japan and the Ryukyu Islands; occurs north of the Miyako Strait from Kyushu to southern Shikoku in open areas, grasslands and sandy habitats below 200 m [12]	Endemic to the Ryukyu Islands; restricted to the Yaeyama Islands south of the Miyako Strait (Yonaguni Island) in open areas, grasslands and sandy habitats below 200 m [35]

* Length × width (mean ± SD)

^abc^ Means in a row without a common superscript letter different (*p* ≤ 0.05; Tukey’s HSD test)

Capitulum width was also narrower in var. *albescens* (1.36 cm, Fig. 5A3) than in *C. brevicaule* (1.79 cm, Fig. 5B3) or *C. irumtiense* (1.93 cm, Fig. 5C3). Its involucral phyllaries averaged only 1.72 mm in width (Fig. 5A4), significantly narrower than those of the other taxa (Fig. 5B, C4). *Cirsium brevicaule* had the highest inner-to-outer phyllary length ratio (2.24, Fig. 5B4), whereas var. *albescens* (1.34, Fig. 5A4) and *C. irumtiense* (1.44, Fig. 5C4) did not differ significantly (Table 1).

Floret traits further separated the three taxa. Corolla color was white in var. *albescens* and *C. brevicaule* (Fig. 5 A, B3, 5) but bluish-purple in *C. irumtiense* (Fig. 5C3, 5). Floret length decreased significantly in the order *C. brevicaule* (2.94 cm, Fig. 5B5) > *C. irumtiense* (2.59 cm, Fig. 5C5) > var. *albescens* (2.36 cm, Fig. 5A5). *Cirsium brevicaule* also had significantly shorter corolla lobes (3.07 mm, Fig. 5B5), whereas var. *albescens* (3.47 mm) and *C. irumtiense* (3.36 mm) did not differ significantly (3.47 mm vs. 3.36 mm, Fig. 5 A, B3, 5).

In summary, var. *albescens* is distinguished by smaller leaves and capitula, hispid leaf surfaces, and narrow phyllaries. In contrast, *C. brevicaule* and *C. irumtiense* share similar overall sizes but differ in floret dimensions, corolla color, and phyllary tiering (Fig. 5, Table 1). These clear morphological differences support treating these taxa as distinct.

### Floral-color polymorphism in *Cirsium japonicum* var. takaoense and its comparison with var. australe

**Fig. 6:**
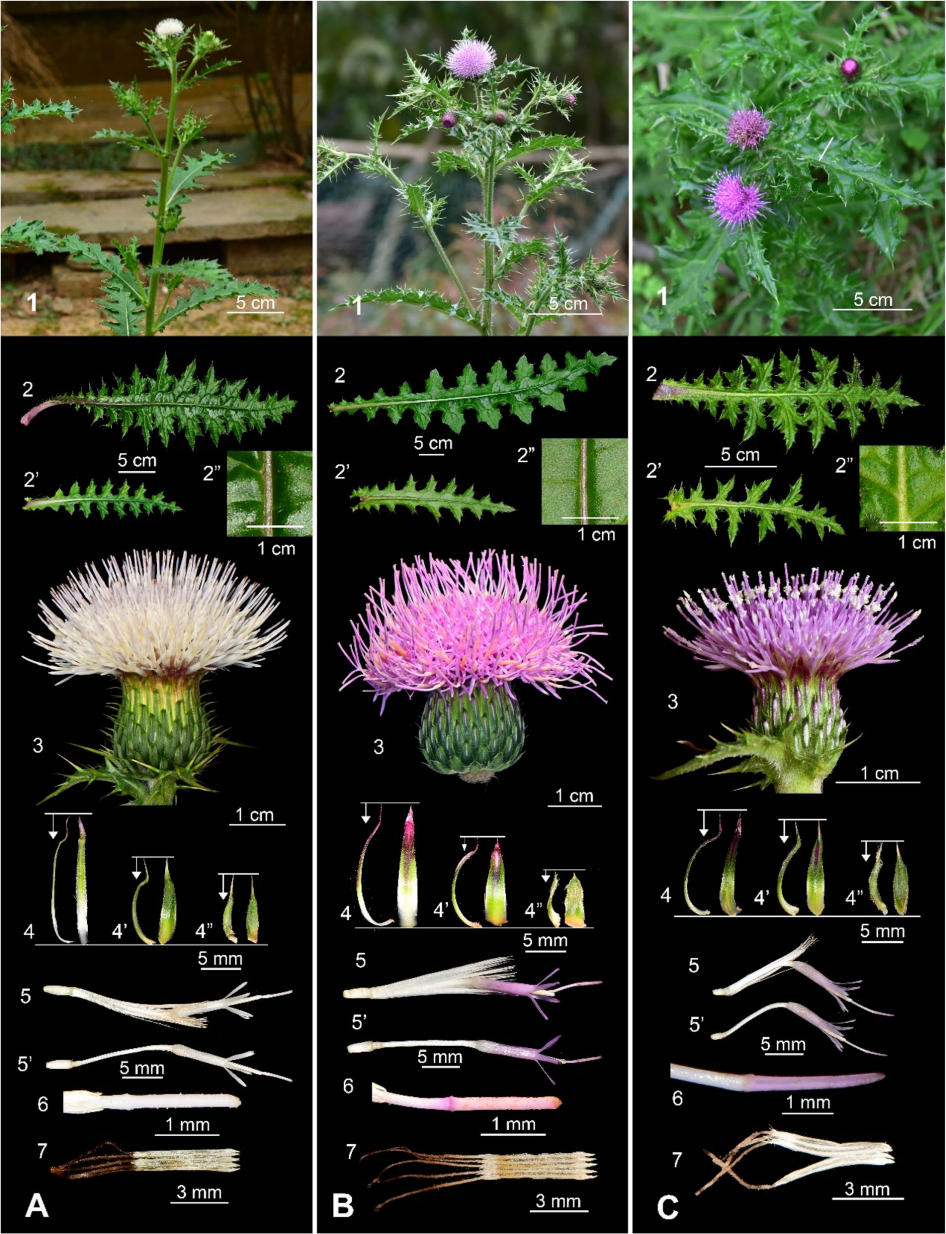
Comparative morphology of white and bluish-purple floral morphs of *Cirsium japonicum* DC. var. *takaoense* Kitam. and var. *australe* Kitam. **A:** var. *takaoense* (white corolla); **B:** var. *takaoense* (bluish‑purple corolla); **C:** var. *australe*. 1: Habit; 2: Rosette leaf; 2’: Cauline leaf; 2″: Adaxial surface 3: Capitula; 4: Inner phyllary; 4’: Middle phyllary; 4″: Outer phyllary; 5: Floret; 5’: Floret (pappus removed); 6: Style branches; 7: Synantherous stamen

To assess morphological differences between var. *australe* and the white and bluish-purple flowered morphs of var. *takaoense*, we measured key vegetative and reproductive traits across multiple populations (Fig. 6, Table 2). Overall, plants of var. *takaoense* (both color forms; Fig. 6 A, B1) were larger than those of var. *australe* (Fig. 6C1), which bears only bluish-purple flowers (Fig. 6C1, 3, 5). Moreover, the mean rosette leaf length of var. *australe* (19.15 cm, Fig. 6C2) was significantly shorter than that in the white (26.99 cm; Fig. 6A2) and bluish-purple (35.26 cm; Fig. 6B2) morphs of var. *takaoense*. The two *takaoense* morphs did not differ significantly from one another, although the bluish-purple form showed a trend toward larger leaves (Table 2). Similarly, cauline leaf length in the bluish-purple morph (22.54 cm; Fig. 6B2’) exceeded both the white morph (14.37 cm; Fig. 6A2’) and var. *australe* (12.66 cm; Fig. 6C2’). The adaxial leaf surfaces of var. *takaoense* ranged from glabrous to sparsely pubescent (Fig. 6 A, B2″), whereas var. *australe* ranged from sparsely to densely pubescent (Fig. 6C2″).

**Table 2:** Morphological comparison of white and bluish-purple floral morphs of *Cirsium japonicum* DC. var. *takaoense* Kitam. and var. *australe* Kitam

Taxon		var. *takaoense* (white corolla)	var. *takaoense* (bluish‑purple corolla)	var. *australe*
Rosette leaves	size (cm)	26.99 ± 10.71^ab^ × 7.86 ± 1.87^b^	35.26 ± 5.85^a^ × 10.73 ± 1.52^a^	19.15 ± 6.26^b^ × 5.92 ± 1.67^b^
	shape	narrowly elliptic to oblanceolate	narrowly elliptic to oblanceolate	narrowly elliptic to oblanceolate
	number of lobe pairs	12 ± 2^a^	15 ± 2^b^	10 ± 2^c^
	surface	glabrous to sparsely pubescent	glabrous to sparsely pubescent	sparsely to densely pubescent
Cauline leaves	size (cm)*	14.37 ± 8.70^b^ × 6.10 ± 3.36^a^	22.54 ± 8.75^a^ × 7.70 ± 2.99^a^	12.66 ± 3.54^b^ × 5.02 ± 1.54^a^
	shape	narrowly elliptic to narrowly triangular	narrowly elliptic to narrowly triangular	narrowly elliptic to narrowly triangular
	number of lobe pairs	8 ± 2^a^	8 ± 2^a^	8 ± 2^a^
	surface	glabrous to sparsely pubescent	glabrous to sparsely pubescent	sparsely to densely pubescent
Capitulum	size (cm)*	3.73 ± 0.54^a^ × 1.43 ± 0.24^a^	3.19 ± 0.72^a^ × 1.26 ± 0.38^a^	3.51 ± 0.82^a^ × 1.56 ± 0.55^a^
	peduncle	conspicuous	conspicuous	inconspicuous
Phyllary	length (cm)	1.14 ± 0.34^a^	1.27 ± 0.80^a^	1.09 ± 0.26^a^
	length ratio (inner vs outer)	2.71 ± 0.47^ab^	2.85 ± 0.73^a^	1.82 ± 0.06^b^
	protrusion (mm)	2.47 ± 0.62^a^	1.35 ± 0.47^a^	2.49 ± 0.54^a^
Floret	length (cm)	3.15 ± 0.35^a^	3.50 ± 0.09^a^	2.38 ± 0.31^b^
	corolla color	white	bluish‑purple	bluish‑purple
	anther length (mm)	6.81 ± 1.25^a^	7.07 ± 0.37^a^	5.64 ± 0.53^b^
	pappus length (cm)	1.71 ± 0.19^a^	1.89 ± 0.04^a^	1.32 ± 0.13^b^
Distribution		Occurs throughout southern Taiwan (including in the Orchid and Green Islands) with scattered populations in central and northeastern regions, in open coastal, plain, and low-mountain habitats below 2,000 m	Occurs in southern Taiwan below 2,000 m elevation in plains or low-mountain open habitats, forest margins, or grasslands	Widely distributed throughout northern and central Taiwan in coastal areas, plains, and low-mountain open habitats, forest margins, or grasslands below 2,000 m elevation. Also found in mainland China and Japan

* Length × width (mean ± SD)

^abc^ Means in a row without a common superscript letter different (*p* ≤ 0.05; Tukey’s HSD test)

Under favorable conditions, var. *takaoense* typically produces a conspicuous peduncle (Fig. 6 A, B1). In contrast, in var. *australe,* cauline leaves usually clasp the capitulum, making the peduncle inconspicuous (Fig. 6C1). In var. *takaoense*, peduncles are inconspicuous under strong wind or nutrient stress. The inner-to-outer phyllary length ratio was greater in the bluish-purple (2.85; Fig. 6B3, 4) and white (2.71; Fig. 6A3, 4) morphs of var. *takaoense* than in var. *australe* (1.82; Fig. 6C3, 4), indicating more pronounced involucre tiering. Var. *australe* consistently bore the smallest florets, anthers, and pappi (Fig. 6C5–7), whereas these floral traits did not differ significantly between the two color morphs of var. *takaoense* (Fig. 6 A, B5–7).

In summary, var. *takaoense* differs from var. *australe*, which bears only bluish-purple flowers, by its larger vegetative size, glabrous to sparsely pubescent leaves, conspicuous peduncles, and more strongly tiered phyllaries (Fig. 6, Table 2). The white and bluish-purple morphs of var. *takaoense* show no structural divergence beyond slight size variation.

## Discussion

### Early sectional splits and downstream lineage divergence

Phylogenomic analyses place subsections *Sinocirsium*, *Arenicola*, and *Nipponocirsium* in three monophyletic clades (PP = 1; Fig. 1), with *Arenicola* sister to *Nipponocirsium*. Divergence time estimates place their split at 1.02 Mya (95% HPD: 0.86–1.18 Mya), coinciding with the pre-Pastonian glaciation (Fig. 2). Subsequent divergences among *C. brevicaule*, *C. irumtiense*, and the three lineages of the *C. japonicum* complex cluster at 0.32–0.44 Mya (95% HPD: 0.23–0.52 Mya), matching the Mindel glaciation. Low sea levels at this time exposed the Taiwan–Ryukyu–mainland shelf, whereas post-Mindel subsidence of the Okinawa–Miyako platform removed this “stepping stone” corridor [4, 6, 8–10]. Ensemble species-distribution models likewise show a southward displacement of suitable habitat for var. *takaoense* at the Last Glacial Maximum, followed by Holocene northward recolonization (Fig. 4). Taken together, these results support a contraction–expansion scenario.

Such early sectional splits form a phylogenetic scaffold, whereas the Taiwanese *C. japonicum* complex represents a much younger, island-centered radiation that diversified following late Pleistocene sea-level oscillations (Fig. 2). Overall, transcriptomic analyses resolve three well-supported lineages (PP = 1; Fig. 1): (i) the Japanese var. *japonicum*; (ii) a Taiwanese white-flower clade comprising var. *albescens* and var. *takaoense*; and (iii) a Taiwanese bluish-purple clade containing var. *australe* and var. *fukienense*. Next, species delimitation tests and genome-size contrasts reveal a clear divergence gradient (Fig. 1, Fig. 3). Importantly, all five taxa share an identical karyotype, 2*n* = 34 [119], so genome-size shifts must reflect changes in DNA content rather than polyploidy. Phylogenetically, var. *japonicum* forms the basal lineage of the *C. japonicum* complex and remains distinct. It retains a genome size of 2 C ≈ 1.20 pg, within the small range reported for *Cirsium* (≤ 1.4 pg [120]) and consistent with the reconstructed ancestral value (~ 1.3 pg). The white-flower pair shows moderate to high support for separation, with var. *takaoense* markedly reduced (2C ≈ 1.01 pg) and var. *albescens* moderately expanded (2C ≈ 1.65 pg). The purple-flower pair displays weaker separation, with var. *australe* and var. *fukienense* both enlarged (2C ≈ 1.79 pg and 1.91 pg, respectively). Collectively, these results indicate that the two Taiwanese clades function as coherent lineages that remain permeable to gene flow, consistent with incipient speciation. Decoupling between chromosome number and genome size in *Helianthus* [121, 122] and *Anacyclus* [123] suggests that DNA-content shifts without karyotypic change recur in the Asteraceae.

Beyond Taiwan, *C. brevicaule* and *C. irumtiense* illustrate how island barriers can intensify divergence. The Miyako Strait forms a sharp phylogeographic break separating two well-supported monophyletic clades (Fig. 1). DensiTree topologies show virtually no recent gene flow between these species (Fig. 1D). The two species also differ sharply in genome size, with *C. brevicaule* at 2 C ≈ 1.93 pg and *C. irumtiense* at 2 C ≈ 0.69 pg (Fig. 4), corroborating their long-standing isolation.

### Reticulate gene flow and corolla polymorphism

Our data resolve all three major lineages, but their recent divergence indicates that reproductive barriers within Taiwan are still developing. As a result, genomic signals of secondary contact and trait polymorphism persist below the sectional scale. Within island lineages, reticulation is pronounced. Neighbor-net (Fig. 1B) and DensiTree (Fig. 1D) analyses place var. *australe* and var. *fukienense* in a densely tangled network. This echoes extensive reticulate patterns reported in North American *Cirsium*, where introgression obscures species boundaries [21, 46].

Corolla color mirrors this reticulation. While the white-flower clade (var. *albescens* and var. *takaoense*) and the bluish-purple clade (var. *australe* and var. *fukienense*) broadly align with phylogeny, color polymorphisms do not. Some var. *takaoense* bear purple corollas, and rare pale-purple forms of var. *fukienense* occur on the Penghu Islands [29]. In contrast, color is uniform within var. *albescens* (white) and var. *australe* (bluish purple). Mismatches between color and phylogeny have been documented in *Iris* (Iridaceae) [124] and *Silene* (Caryophyllaceae) [125] but remain unexplored in East Asian Compositae.

Given these patterns, we provisionally retain the four Taiwanese entities at varietal rank, a decision that reflects diagnosable morphology, near-monophyly in transcriptomic trees, and lineage-specific genome-size shifts. In contrast, the fact that var. *japonicum* [41] has a wide geographic range suggests that it may merit elevation, pending denser sampling and integrative evidence. Future work should dissect the genetic basis of color polymorphism and test whether variation arises from introgression, parallel mutation, or ecological selection.

### Niche divergence drives genome size–climate interactions in Taiwanese *Cirsium japonicum* varieties

We found that divergent DNA content correlates with contrasting ecological niches among the Taiwanese varieties. Within the *C. japonicum* complex, var. *takaoense* and var. *fukienense* display opposing genome-size trajectories (Fig. 3) and occupy broad, largely nonoverlapping ranges (Supplementary Figure S4). Because these two varieties belong to different phylogenetic lineages, parallel demographic patterns are unlikely to reflect a shared ancestral bottleneck. Competitive exclusion or distinct environmental filters therefore remain plausible explanations [126, 127].

Model response curves highlight these differences (Supplementary Table S5). For var. *takaoense*, mean diurnal range (BIO2, 61.7%) is the strongest predictor. Small genomes are often associated with faster cell cycles, reduced nucleic acid costs, and accelerated early growth, traits that facilitate rapid use of favorable periods under variable temperatures [128–130]. Consistently, var. *takaoense* attains a larger plant size than its congeners (Fig. 6, Table 2), a pattern also reported in *Ambrosia artemisiifolia* (Compositae) [129] and *Phalaris arundinacea* (Poaceae) [128]. At the same time, small genomes can be linked to r-selected life-history traits such as high fecundity and short generation times.

By contrast, warm-season precipitation (BIO18, 77.1%) is the strongest predictor for var. *fukienense*. Genome expansion is frequently associated with greater drought and salt tolerance [131–133]. Although many examples involve polyploids, such as *Pugionium* (Brassicaceae) [132] and *Jasione maritima* (Campanulaceae) [133], diploid cases are also known. For instance, hybrid-derived *Helianthus deserticola* (2*n* = 34) has a genome roughly 50% larger than that of its progenitors and exhibits improved survival under desert drought [134, 135]. Similarly, larger genomes occur in certain *Quercus* species from regions with strongly seasonal precipitation [136].

Taken together, these findings suggest that genome-size divergence alone does not dictate demographic fate. Rather, interactions between genome-mediated physiology and late Pleistocene to Holocene climate change have produced contrasting population histories: a climate-sensitive, small-genome lineage prone to boom–bust cycles, and a large-genome lineage buffered against drought yet confined to shifting coastal refugia. We consider the deeper context of these contrasts in the following section.

### Land bridges, terminal isolation, and paleoclimate-modulated demographic rebounds

During late Pleistocene low stands, the Taiwan–Ryukyu continental shelf emerged as a broad “stepping-stone” corridor. Furthermore, after post-Mindel subsidence (~ 0.4 Mya) reinstated deep straits [4, 6, 8–10], isolation timing became strongly correlated with dispersal ability. Although gravity-dispersed trees (e.g., *Camellia japonica*) diverged first (~ 1.8 Mya [137]), wind-dispersed Compositae, including the *Cirsium* pair and *Ixeridium* [138] became fully isolated in the mid-Pleistocene (0.5–0.3 Mya), while bird-dispersed Lauraceae (e.g., *Neolitsea sericea*) show the youngest isolation date (< 0.1 Mya [139]).

Island fragmentation has also promoted diversification within *Cirsium*. For example, *Cirsium brevicaule* and *C. irumtiense* are separated by the Miyako Strait and display a 2.8-fold genome-size disparity (Fig. 3). Although Yonaguni, the westernmost Ryukyu Island, lies only 108 km from Taiwan [140], neither *C. brevicaule* nor *C. irumtiense* occur in Taiwan or its outlying islets. End-of-arc isolation has therefore amplified divergence, and the terminal Ryukyu islands now harbor deeply differentiated populations of *C. brevicaule* on Amami Ōshima and *C. irumtiense* on Yonaguni (Fig. 1). Furthermore, SODA analysis (Fig. 1C) delimits both island groups as separate evolutionary entities. These island isolates are morphologically indistinguishable from other conspecifics. However, their deep genetic distances underscore the biogeographic impact of terminal-arc isolation and suggest incipient speciation or strong local adaptation. Moreover, although combined effects of tectonic subsidence in the Okinawa Trough and subsequent interglacial sea-level rises may have ultimately severed gene flow between the Ryukyu Arc and Taiwan [141, 142], finer-scale processes have continued to shape genetic diversity within remaining island refugia.

We then reconstructed demographic histories of two Taiwanese varieties from the Late Pleistocene into the Holocene. Our EBSP and SDM results suggest a delayed bottleneck in var. *takaoense*: gradual decline through the last glacial, sharp contraction near the LGM (~ 21 kya), reaching a minimum that coincides with the Younger Dryas (12.9–11.7 kya), and slow rebound thereafter (Fig. 4). Two factors may help account for this delay. First, after the LGM, recolonization may have been slowed by limited dispersal, priority effects, and rapid forest infilling of former open slopes [143, 144]. Second, the Younger Dryas cooling (12.9–11.7 kya) could have briefly narrowed the climatic window for var. *takaoense*, potentially postponing its rebound until early-Holocene warming [145, 146].

In contrast, var. *fukienense* shows a nearly flat EBSP despite SDMs predicting wide shelf habitats at low sea levels (Fig. 4B), indicating that its saline, wind-exposed coastal niche simply shifted landward with the retreating shore (Supplementary Figure S4). Pronounced Younger Dryas climate volatility may therefore have turned the small-genome advantage into a transient bust [130, 147, 148], whereas the large-genome coastal lineage appears to have remained buffered yet spatially constrained [131–133].

Taken together, these results outline a three-step biogeographic narrative that may involve (i) an initial land-bridge connection, (ii) subsequent island-arc fragmentation, and (iii) a paleoclimate-modulated demographic rebound. These phases provide a plausible explanation for the structured diversification observed across the Taiwan–Ryukyu region.

### Revised taxonomic boundaries and implications for medicinal resource management

Morphometric and molecular evidence supports treating *C. japonicum* var. *albescens*, *C. brevicaul*e, and *C. irumtiense* as three distinct species (Fig. 1, Fig. 5; Table 1), while identifying var. *takaoense* (both color forms) and var. *australe* as incipient independent lineages. Regional floras and conservation lists should be updated to reflect these changes.

Moreover, conservation and patterns of use are often closely linked. *Cirsium japonicum* var. *albescens* is confined to Taiwan’s Hengchun Peninsula [11, 33] and is harvested for medicine [149], making it vulnerable to habitat loss and overharvesting [150]. Accurate identification underpins pharmacological screening and supply-chain traceability [151, 152]. Finally, since all five taxa occur at low elevations, large-scale cultivation is feasible if provenance is documented and locally adapted germplasm is maintained [150, 153].

### Taxonomic revision of *Cirsium brevicaule* and allied taxa

Earlier treatments beginning with Kitamura [25] placed four taxa, *C. albescens* (≡ *C. japonicum* var. *albescens*), *C. morii*, *C. brevicaule*, and *C. irumtiense*, in subsect. *Arenicola* [12, 25, 154]. Kitamura reduced *C. irumtiense* to a variety of *C. brevicaule* [154], and Kadota later treated them as synonyms [12], but our phylogenetic analyses reached a different conclusion (Fig. 1). The trees resolve *C. brevicaule* and *C. irumtiense* as reciprocally monophyletic clades separated by the Miyako Strait, and neighbor-Net analysis underscores their deep genetic divergence (Fig. 1B). Our morphological data are congruent (Fig. 5, Table 1): *C. irumtiense* has purple corollas and densely pubescent leaves, whereas *C. brevicaule* has white corollas and nearly glabrous foliage [35]. Species-delimitation tests likewise support their recognition as independent evolutionary units (Fig. 1C).

Within *C. brevicaule*, SODA isolates the Amami Ōshima population (historical *C. brevicaule* var. *oshimense* [154]); within *C. irumtiense*, it likewise separates the Yonaguni population. We retain these island isolates as evolutionarily significant units for two reasons. First, stabilizing selection coupled with phenotypic plasticity maintains capitulum size, phyllary architecture, and leaf armature near a shared adaptive optimum across salt-sprayed coastal grasslands, masking genomic divergence [155, 156]. Second, occasional long-distance seed dispersal via the plumose pappus can homogenize alleles at adaptive loci while allowing the remainder of the genome to diverge [157]. The slightly higher abaxial-midrib trichome density in var. *oshimense* [154] falls within the range observed elsewhere in *C. brevicaule* and thus lacks diagnostic value. No fixed phenotype separates the Amami and Yonaguni isolates, so naming them would fail the diagnosability criterion. Genome-size data (2C ≈ 1.93 pg in *C. brevicaule* vs. 0.69 pg in *C. irumtiense*; Fig. 3) nevertheless corroborate their long-standing isolation and species status.

Specimens from Taiwan’s Hengchun Peninsula now called *C. albescens* [33] were also once referred to *C. brevicaule* [12, 31], yet they fall firmly within the *C. japonicum* complex, far from subsect. *Arenicola* (Fig. 1). These plants have scabrous, hispid leaves, smaller capitula, profuse branching, and inconspicuous peduncles [11, 31, 33, 154], distinguishing them from both Ryukyuan taxa (Fig. 5, Table 1). Within the *C. japonicum* complex, they are sister to var. *takaoense* (Fig. 1, Fig. 6 A, B) but differ in habit, indumentum, and distribution, being restricted to southern Taiwan [11, 31, 35]. Misidentifications arose from superficial resemblance rather than close affinity; the taxon is best maintained as *C. japonicum* DC. var. *albescens* (Kitam.) Y.H.Tseng, P.C.Liao & Chih Y.Chang.

Although *C. morii* was originally assigned to subsect. *Arenicola* because of its broad phyllaries [12, 25, 154], molecular data place it at the base of subsect. *Nipponocirsium* (Fig. 1). This species combines unusual traits for that clade, including large leaves on both rosette and flowering stems, sparse cobwebby indumentum, and well-developed stolons [11, 12, 25, 154]. Its rosette-bolting form contrasts with the cauline-leaf habit predominant elsewhere in *Nipponocirsium* [26, 28], suggesting retention of ancestral features. Consistent recovery across delimitation methods indicates a distinct lineage (Fig. 1C), though broader sampling is needed before proposing a separate subsection.

In summary, subsect. *Arenicola* is restricted to *C. brevicaule* and *C. irumtiense*. Taiwan has no native members of that subsection; *C. albescens* belongs to the *C. japonicum* complex, and *C. morii* is an early-diverging member of subsect. *Nipponocirsium*.

### Taxonomic revision of the *Cirsium japonicum* complex

Early East Asian floras followed Gray [158] and Jones [159] in placing all thistles in *Cnicus* [19, 21], but after Greene [160] revised its circumscription, *Cirsium* became the accepted name from the 1910 s onward [161, 162]. Taxonomic confusion persisted in the *C. japonicum* complex of Taiwan and nearby regions. Misidentifications involving *C. brevicaule*, var. *japonicum*, var. *australe*, and var. *fukienense* were common: Forbes and Hemsley [163] and Henry [164] recorded *Cnicus japonicus* (= *C. japonicum*) from Taiwan, but these specimens correspond to var. *australe* [25, 154], and both var. *albescens* and var. *fukienense* were often assigned to *C. brevicaule* (= *Cnicus brevicaulis*) [161, 165].

Li [166] recognized a native Taiwanese var. *japonicum*, and Peng [11] treated it as var. *australe*, but both authors’ specimens belong to extant Taiwanese lineages. Our phylogenetic analyses (Fig. 1A, E) show Japanese var. *japonicum* as substantially divergent from the Taiwanese clade; among Taiwanese lineages, var. *australe* is closest to var. *japonicum* (Fig. 1B). Both are widespread in East Asia but occupy opposite sides of a latitudinal break at ~ 30°–33° N, with var. *australe* to the south and var. *japonicum* to the north [41]. Broader sampling is needed to clarify relationships across this range.

Traditional distinctions based solely on corolla color, white for var. *takaoense*, bluish purple for var. *australe* [29, 31, 167], do not match evolutionary relationships (Fig. 1A, B). Apart from var. *albescens*, white- and bluish-purple-corolla groups form a paraphyletic assemblage within a strongly supported clade characterized by greater stature, shallower leaf lobes, shorter phyllary spines, and conspicuous peduncles (Fig. 6A, B, Table 2). Morphological comparisons with var. *australe* therefore justify uniting central–southern white- and bluish-purple-corolla populations under var. *takaoense*.

Although Kitamura [40] reduced var. *fukienense* to forma rank (*C. japonicum* DC. f. *fukienense* (Kitam.) Kitam.), our phylogenetic results (PP = 1; Fig. 1A, E), species-delimitation signal (Fig. 1C), and its distinctive long, spine-tipped phyllaries all support retaining it at varietal rank [29]. For clarity, we retain the name var. *fukienense*.

In summary, our evidence resolves long-standing taxonomic ambiguity within the *C. japonicum* complex. Japanese var. *japonicum* is distinct and absent from Taiwan; central–southern Taiwanese white- and bluish-purple-corolla populations form a single lineage best treated as var. *takaoense*; var. *fukienense* clearly warrants retention at varietal rank; var. *australe* remains the widespread purple-flowered taxon; and the endemic var. *albescens* is restricted to the Hengchun Peninsula. This framework will support future ecological, pharmacological, and conservation research on East Asian thistles.

### Taxonomic information

#### Key to the *Cirsium japonicum* complex (subsect. *Sinocirsium*) and related taxa of subsect. *Arenicola* in Taiwan and the Ryukyu Islands

1. Phyllaries broad, > 2 mm wide, prominent phyllary apices flat and obtuse (Subsect. I. Arenicola):

2. Corollas white; involucre with pronounced tiering; leaves glabrous to shortly pubescent .....1.* C. brevicaule*

2. Corollas bluish‑purple; involucre weakly tiered; leaves pubescent ...... 2. * C. irumtiense*

1. Phyllaries narrow, < 1.9 mm wide; prominent phyllary apices conical and spine-like (Subsect. II. Sinocirsium):

3. Phyllary apical spine longer than 3 mm; inner and outer phyllaries similar in length:

4. Corollas all white; leaves hispid ....... 3a. *C. japonicum* var. *albescens*

4. Corollas bluish-purple or less light purple; leaves pubescent or hirsute ...... 3d. *C. japonicum* var. *fukienense*

3. Phyllary apical spine is often shorter than 2 mm; inner and outer phyllaries have different lengths:

5. Leaves hirsute; peduncle not obvious; corollas bluish purple ...... 3c. *C. japonicum* var. *australe*

5. Leaves smooth; peduncle obvious; corolla white or bluish purple ...... 3b. *C. japonicum* var. *takaoense*

### Taxonomic treatment

Integrating the foregoing results and discussion, we present the following taxonomic treatments focused on Taiwan and the Ryukyu Islands. Although several lineages recovered in this study show clear genetic and morphological distinctiveness, including lineages corresponding to *C. japonicum* var. *japonicum*, we refrain from proposing additional rank changes here, given the geographically focused sampling of the present study.

Additional comparative images of the remaining taxa are provided in Supplementary Figure S5, and complete lists of the specimens examined are provided in Supplementary Table S6. Typification follows the ICN (Shenzhen Code) [168], with treatment of pre-1958 names following the principles summarized by McNeill [169], especially concerning the application of Articles 9 and 40.

Subsect. I. *Arenicola* Kitam. in Acta Phytotax Geobot. 3: 3. 1934.

1. *Cirsium brevicaule* A.Gray, Mem. Amer. Acad. Arts. Sci. 6: 396. 1859.

- ≡ *Cnicus japonicus* var. *brevicaulis* (A.Gray) Maxim., Mel. Biol. 9: 324. 1874.

Type: JAPAN. “Loo-Choo Island” [Ryukyu], *C. Wright s. n.* (lectotype: HUH 6012!, designated here, Supplementary Figure S6; isolectotype: HUH 6010!, Supplementary Figure S7).

Diagnosis: Readily distinguished from allied taxa by its white corollas, clearly tiered involucre, and flat, obtuse phyllary apices. Leaves are glabrous to shortly pubescent, and the phyllaries are characteristically broader than in the *C. japonicum* complex, typically exceeding 2 mm in width.

Distribution: Confined to the central Ryukyu Arc, from Amami Ōshima south to Okinawa Hontō and nearby islets [154]. Absent from Taiwan, the Yaeyama Islands, and the Asian mainland. Occupies coastal slopes and disturbed grasslands below ca. 200 m, typically on calcareous or gravelly substrates. Historical Taiwanese records are misidentified specimens of the *C. japonicum* complex (most often var. *albescens*) [11, 31, 161, 164, 165]; true *C. brevicaule* is endemic to the Ryukyus.

Conservation status: The species spans many islands from Amami Ōshima to Okinawa, giving an extent of occurrence > 20,000 km^2^ and > 20 locations. Thrives in disturbed coastal grasslands, some within protected areas. No evidence of decline or major threats. Following [170], assessed as Least Concern (LC).

2. *Cirsium irumtiense* Kitam., Acta Phytotax. Geobot. 2(1): 41. 1933.

- ≡ *Cirsium brevicaule* A.Gray var. *irumtiense* (Kitam.) Kitam., Mem. Coll. Sci. Kyoto Imp. Univ., Ser. B, Biol. 13: 59. 1937.

Type: JAPAN. “Liukiu” [Ryukyu], “Insula Irumti” [Iriomote Island], July 1923, *G. Koidzumi s. n.* (cited as type at KYO).

Diagnosis: Separated from *C. brevicaule* by its bluish-purple corollas, a weakly tiered involucre, and more densely pubescent leaves.

Distribution: Confined to the southern Ryukyu Arc, throughout the Yaeyama Islands [35]. Verified on Iriomote-jima (type locality), Ishigaki-jima, Miyako-jima, and Yonaguni-jima. Northern limit is the Miyako Strait, beyond which its sister species *C. brevicaule* occurs. Found below 200 m on coastal grasslands, roadside embankments, and lightly disturbed maritime slopes, usually on limestone or gravelly substrates exposed to salt spray.

Conservation status: Occurs across the Yaeyama Islands, including > 10 small islets. Extent of occurrence ca. 600 km^2^, > 20 locations. Tolerates disturbance; some populations within protected areas. No evidence of decline or major threats. Following [170], assessed as LC.

Subsect. II. *Sinocirsium* Kitam., Acta Phytotax. Geobot. 3(1): 3. 1934.

3. *Cirsium japonicum* DC., Prodr. [A. P. de Candolle] 6: 640. 1838.

3a. var. *albescens* (Kitam.) Y.H.Tseng, P.C.Liao & Chih Y.Chang, *comb. nov.*

- *≡ Cirsium albescens *Kitam., Acta Phytotax. Geobot. 1(1): 56. 1932.
- *= Cirsium brevicaule auct. *non A.Gray: Peng, Flora of Taiwan 4: 904. 1998; Peng & Chung, Man. Taiwan Vasc. Pl. 4: 244. 2000.
- *= Cirsium maritimum auct. *non Makino: Sasaki, Natu. Hist. SoC. Formos. 406. 1928.

Type: TAIWAN. “Garanbi” [Eluanbi], Takao, 18 Feb. 1932, *S. Kitamura F-1020* (lectotype: TI!, designated here, Supplementary Figure S8; isolectotype: TAI 110704!, Supplementary Figure S9).

Diagnosis: Distinguished by its all-white corollas, hispid leaves, and long phyllary apical spines (> 3 mm). Plants are often smaller in stature, and the inner and outer phyllaries are similar in length and conspicuously robust, a combination that differs from most other varieties of *C. japonicum*.

Distribution: Narrow endemic on the eastern Hengchun Peninsula, Taiwan, from sea level to ca. 300 m in coral-limestone grasslands, roadside banks, scrub margins, and fallow fields.

Conservation status: Formerly assessed as *C. brevicaule* (LC) [171, 172], now recognized as *C. japonicum* var. *albescens*. Range limited to the eastern half of Hengchun Peninsula; habitat fragmented into few discrete sites. Despite partial occurrence within Kenting National Park, threats include medicinal harvesting and loss of grassland to succession and brush encroachment. Area of occupancy < 500 km^2^ with ongoing habitat decline; meets IUCN criteria [170] for Vulnerable (VU B2b(ii, iii)).

3b. var. *takaoense* Kitam., Acta Phytotax. Geobot. 1(1): 57. 1932.

- ≡ *Cirsium japonicum* DC. var. *australe* Kitam. f. *takaoense* (Kitam.) Yamamoto, Journ. Trop. Agric. 8(1): 277. 1936.
- = *Cirsium lidaoens* S.S.Ying, New Taxa New Names 7: 564. 2024, *syn. nov.*
- *Cirsium formosanum* Sasaki, Inst. Gov. Res. Formos. 9: 507. 1930, *nom. nud.*

Type: TAIWAN. Takao, Mt. Daijurin, 23 Feb. 1932, *S. Kitamura 71,209* (lectotype: KYO 21951!, designated here, Supplementary Figure S10).

Diagnosis: Recognized by its short phyllary apical spines (< 2 mm), inner and outer phyllaries of unequal length, glabrescent leaves, and a clearly developed peduncle. Corollas are white or bluish-purple. Compared with var. *australe*, the leaves are less hirsute, typically broader, and the leaf lobes less pronounced.

Distribution: Southeastern mainland China and Taiwan. In Taiwan, occurs from sea level to ca. 2,000 m in coastal dunes, alluvial plains, foothills, and lower montane slopes. Common in central–southern and southeastern districts, eastern areas, and on Orchid (Lanyu) and Green (Lyudao) Islands; scattered in central-western foothills. Habitats include coastal grasslands, river terraces, fallow fields, and forest margins.

Conservation status: LC [171, 172].

Note: *Cirsium lidaoense* is treated here as a synonym of *C. japonicum* var. *takaoense*. Comparative morphological observations and phylogenomic placements indicate that the population geographically proximate to the type locality (e.g., specimen 3835; Fig. 1) falls within the variation of var. *takaoense* and shows no distinguishing characters.

3c. var. *australe* Kitam., *Cirs*. Nov. Orient.-Asiat. 12. 1931; Yamamoto in Journ. Trop. Agric. 8(1): 277. 1936.

- = *Cnicus japonicus* Maxim., Forbes & Hemsley, Journ. Linn. Soc. 23: 461. 1888; Henry, List Pl. Formos. 55. 1896; Hayata, Comp. Formos. 27. 1904.
- = *Cirsium japonicum* DC., Hayata, Icon. Pl. Formos. 8: 70. 1919.
- = *Cirsium japonicum* DC. var. *japonicum* auct. non DC.: Li, Fl. Taiwan 4: 833. 1978.
- = *Cnicus brevicaulis* auct. non A.Gray: Hayata, Comp. Formos. 27. 1904.
- = *Cirsium brevicaule* auct. non A.Gray: Hayata, Icon. Pl. Formos. 8: 70. 1919.
- = *Cirsium pitouchaoense* S.S.Ying, New Taxa New Names 3: 236. 2022, *syn. nov.*

Type: TAIWAN. Tamsui, Taihokusui, 1914, *U. Faurie 830* (lectotype: KYO 21949!, designated here, Supplementary Figure S11; isolectotype: KYO 21950!, Supplementary Figure S12); Takaoshu: Ubique communis, 1903, *U. Faurie 223* (paratype: KYO20457!).

Diagnosis: Distinguished by its short phyllary apical spines (< 2 mm), inner and outer phyllaries of unequal length, hirsute leaves, and an often poorly developed peduncle. Corollas are bluish-purple throughout. Leaf lobes are typically deeper and more pronounced than in var. *takaoense*, but less deeply developed than in var. *japonicum* (Supplementary Figure S5C), and the capitula are usually more robust than in var. *japonicum* (Supplementary Figure S5A, B).

Distribution: Southern representative of the *C. japonicum* complex. Widely distributed in warm-temperate to subtropical East Asia, south of the 30°–33° N limit of var. *japonicum* [41]. In Taiwan, ranges from coastal flats and plains to lower montane slopes, frequent in central and northern districts from sea level to ca. 1,800 m [29]. Found in coastal grasslands, forest margins, roadside embankments, fallow terraces, and disturbed grassy slopes.

Conservation status: LC [171, 172].

Note: *Cirsium pitouchaoense* is treated here as a synonym of *C. japonicum* var. *australe*. Comparative morphological observations and phylogenomic placements indicate that the population geographically proximate to the type locality (e.g., specimen 3721; Fig. 1) falls within the variation of var. *australe* and shows no distinguishing characters.

3d. var. fukienense Kitam., Acta Phytotax. Geobot. 1(2): 149. 1932.

- ≡ *Cirsium japonicum* DC. f. *fukienense* (Kitam.) Kitam., Acta Phytotax. Geobot. 41(4–6): 179. 1990.
- = *Cnicus brevicaulis* auct. non A.Gray: Henry, List Pl. Formos. 55 (1896); Hayata, Comp. Formos. 27. 1904.

Type: CHINA. Fujian Province, Mt. Wushishan (烏石山), 28 June 1910, *S. Nagasawa s. n.* (lectotype: KYO 21952!, designated here, Supplementary Figure S13).

Diagnosis: Characterized by long phyllary apical spines (> 3 mm), bluish-purple to pale-purple corollas, and pubescent to hirsute leaves (Supplementary Figure S5D–F). Inner and outer phyllaries are similar in length, differing from var. *australe* and var. *takaoense*. Foliar vestiture is typically stronger than in var. *albescens*.

Distribution: Southeastern mainland China (Fujian Province [36, 40]) and Taiwan [29]. In Taiwan, occurs in Yangmingshan National Park (north) and on the offshore archipelagos of Penghu, Kinmen, and Matsu at 0–400 m. Found in coastal grasslands, forest margins, and disturbed grassy slopes.

Conservation status: Large, stable populations on multiple offshore islands and in northern Taiwan; also in Fujian Province, China. No documented threats. IUCN category: LC [170].

## Conclusion

In this paper we combined phylotranscriptomic trees, genome-size trajectories and paleodemographic models, to resolve East Asian *Cirsium* spp. into two Ryukyu species (*C. brevicaule* and *C. irumtiense*) and a five-lineage *C. japonicum* complex that spans from Taiwan to Japan. We conclude that Quaternary glaciations, repeated island fragmentation, and independent shifts in 2 C values emerged as the primary drivers of this radiation. In contrast, late Pleistocene bottlenecks and Holocene expansions explain the present-day population structure.

Furthermore, conservation attention should now focus on the Hengchun endemic *C. japonicum* var. *albescens*, which is vulnerable, and on genome-informed germplasm banking for all lineages that are subjected to medicinal harvest. We also clarify the anomalous prior placement of *C. morii*, extend population sampling of widespread varieties *japonicum* and *australe*, and provide an experimental linkage of the floral-color polymorphism of var. *takaoense* to anthocyanin gene expression and pollinator preference. Taken together, we believe that these steps will refine our understanding of the adaptation and biogeography of *Cirsium* spp. while supporting its sustainable use.

## Supplementary Information

Supplementary Material 1. 

## Data Availability

Raw sequence reads have been submitted to the Sequence Read Archive (SRA) of the National Center for Biotechnology Information (NCBI) under BioProject ID PRJNA1311153. These data are available in read-only format at the following link: [https://www.ncbi.nlm.nih.gov/bioproject/PRJNA1311153](https:/www.ncbi.nlm.nih.gov/bioproject/PRJNA1311153).

## Abbreviations

AUC: Area under the ROC curve
CP: Calibration points
DEM: Digital elevation model
EBSP: Extended Bayesian skyline plot
FRPS: Flora Reipublicae Popularis Sinicae
GMYC: Generalized mixed Yule coalescent
HPD: Highest posterior density
LC: Least Concern
LGM: Last Glacial Maximum
NCBI: National Center for Biotechnology Information
OG: Orthologous gene
OTU: Operational taxon units
PP: Posterior probability
SDM: Species distribution modeling
