# Supplementary material for: Phylotranscriptomics and genome-size evidence clarify the Taiwanese Cirsium japonicum complex and delimit C. brevicaule and allied East Asian thistles
Source: BMC Plant Biol. 2026 Feb 5;26:545. doi: 10.1186/s12870-026-08097-6 (PMC13020037; doi:10.1186/s12870-026-08097-6)
Supplement: Supplementary file 1 — Supplementary Material 1. [file 12870_2026_8097_MOESM1_ESM.docx]

Phylotranscriptomics and genome-size evidence clarify the Taiwanese *Cirsium japonicum* complex and delimit *C. brevicaule* and allied East Asian thistles

Chih-Yi Chang^1^, Pei-Chun Liao^2^, Hsy-Yu Tzeng^3^, Junko Kusumi^4^, Zhi-Hui Su^5,6^, Yen-Hsueh Tseng^1, 3*^

1 Taiwan Forestry Research Institute, No. 53, Nanhai Rd., Zhongzheng Dist., Taipei City, 10066, Taiwan

2 School of Life Science, National Taiwan Normal University, No. 88, Sec. 4, Ting-chow Rd., Wenshan Dist. 116, Taipei City, Taiwan

3 Department of Forestry, National Chung Hsing University, No. 145, Hsing Ta Rd, 402, Taichung City, Taiwan

4 Department of Environmental Changes, Faculty of Social and Cultural Studies, Kyushu University, Fukuoka 819-0395, Japan

5 JT Biohistory Research Hall, Takatsuki, Osaka 569-1125, Japan.

6 Department of Biological Sciences, Graduate School of Science, Osaka University, Osaka 560-0043, Japan

* Corresponding author: Yen-Hsueh Tseng ([tsengyh2014@tfri.gov.tw](mailto:tsengyh2014@tfri.gov.tw))

# Supplementary material

## Supplementary Figure


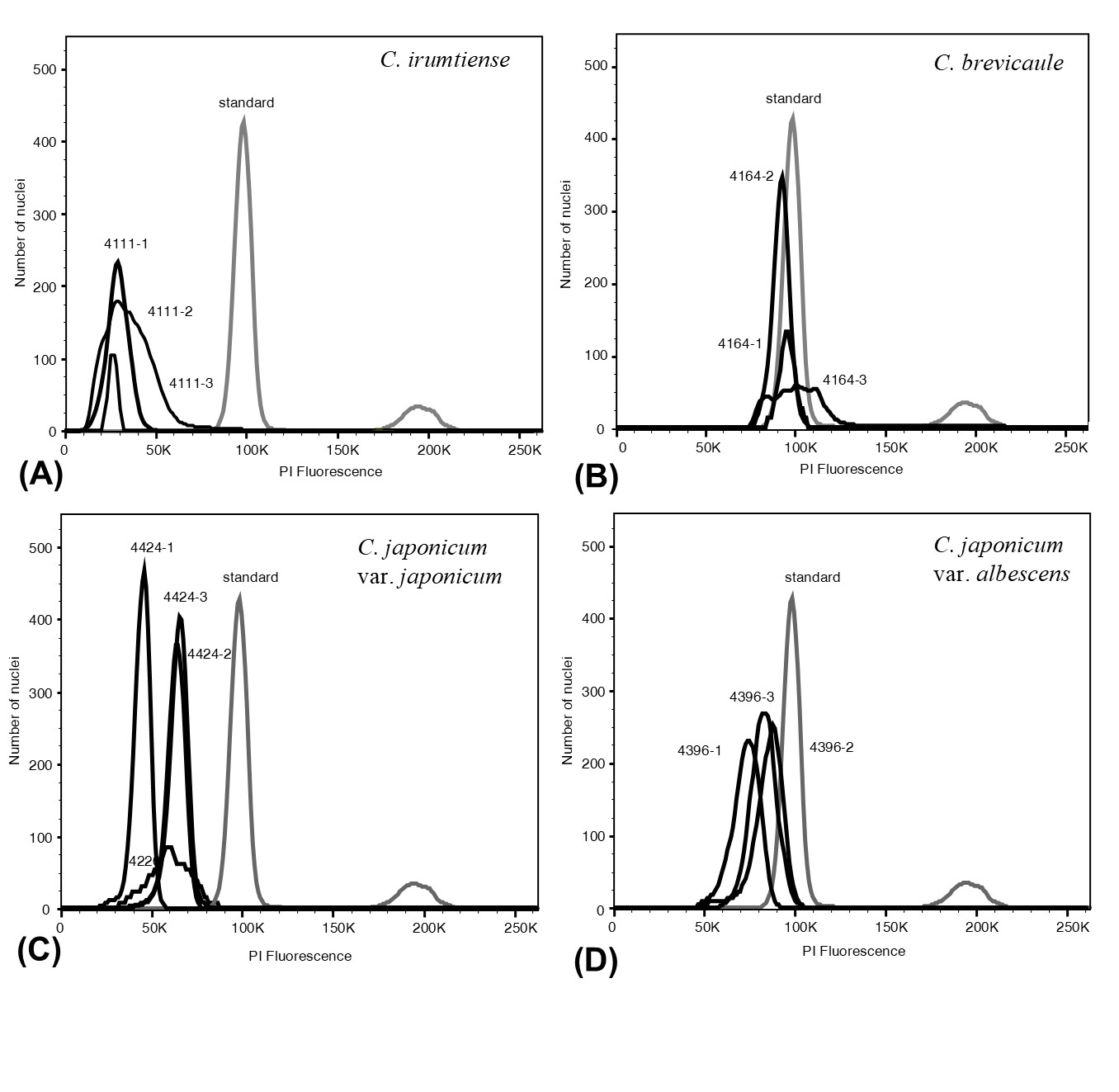


Supplementary Figure S1. Flow cytometric histograms used to estimate genome size (2C) in *Cirsium* taxa.

(A) *C. irumtiense*; (B) *C. brevicaule*; (C) *C. japonicum* var. *japonicum*; (D) *C. japonicum* var. *albescens*. Each sample (black) was cochopped and analyzed together with the internal reference standard *Solanum lycopersicum* (gray, 2C = 2.0 pg).


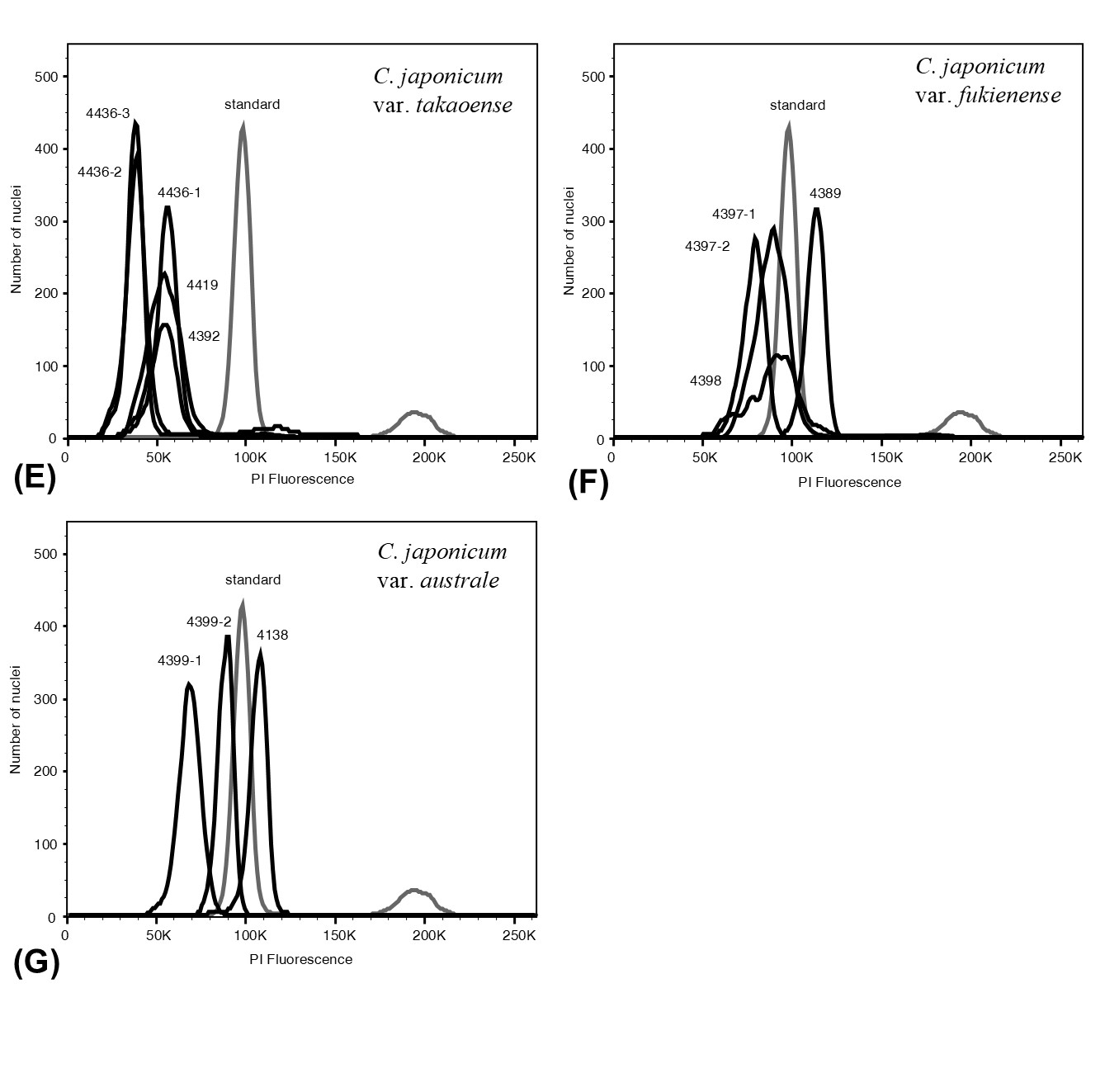


Supplementary Figure S1. (continue)

(E) *C. japonicum* var. *takaoense*; (F) *C. japonicum* var. *fukienense*; (G) *C. japonicum* var. *australe*. Each sample (black) was cochopped and analyzed together with the internal reference standard *Solanum lycopersicum* (gray, 2C = 2.0 pg).


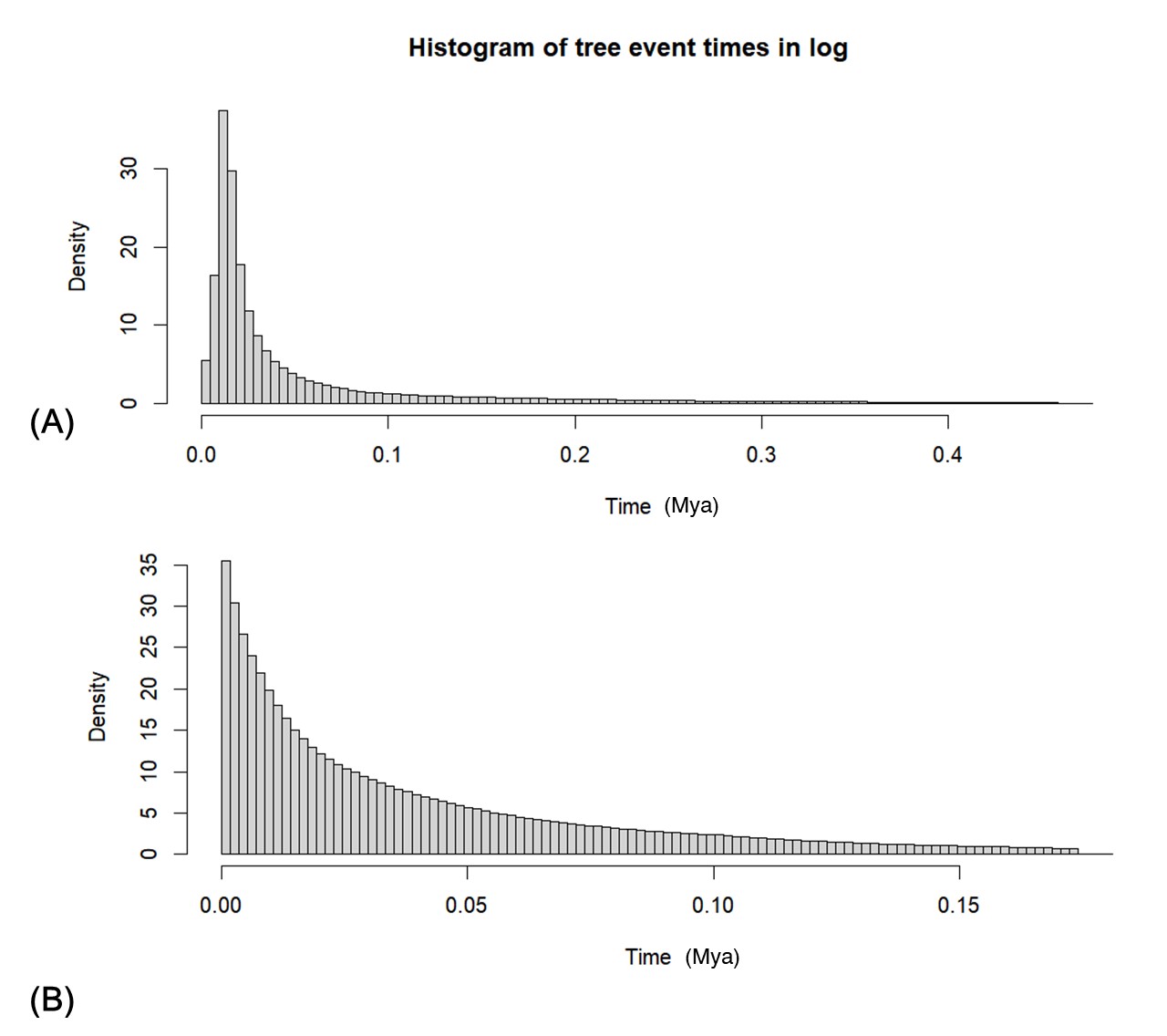


Supplementary Figure S2. Histograms of tree event times for two varieties of *Cirsium japonicum:* **(A)** var. *takaonense* and **(B)** var. *fukienense*. Most coalescent events are concentrated within the last 0.1 million years ago (Mya), with few events occurring beyond this time. This suggests that demographic inferences from the Skyline analysis after 0.1 Mya may have been shaped primarily by the prior rather than by empirical data; thus, interpretation is focused on the period within 0.1 Mya.

Supplementary Figure S3. Hierarchical clustering analysis of Pearson correlations among bioclimatic variables. Branch colors represent groups of variables with absolute Pearson correlation coefficients (|r|) exceeding 0.8.


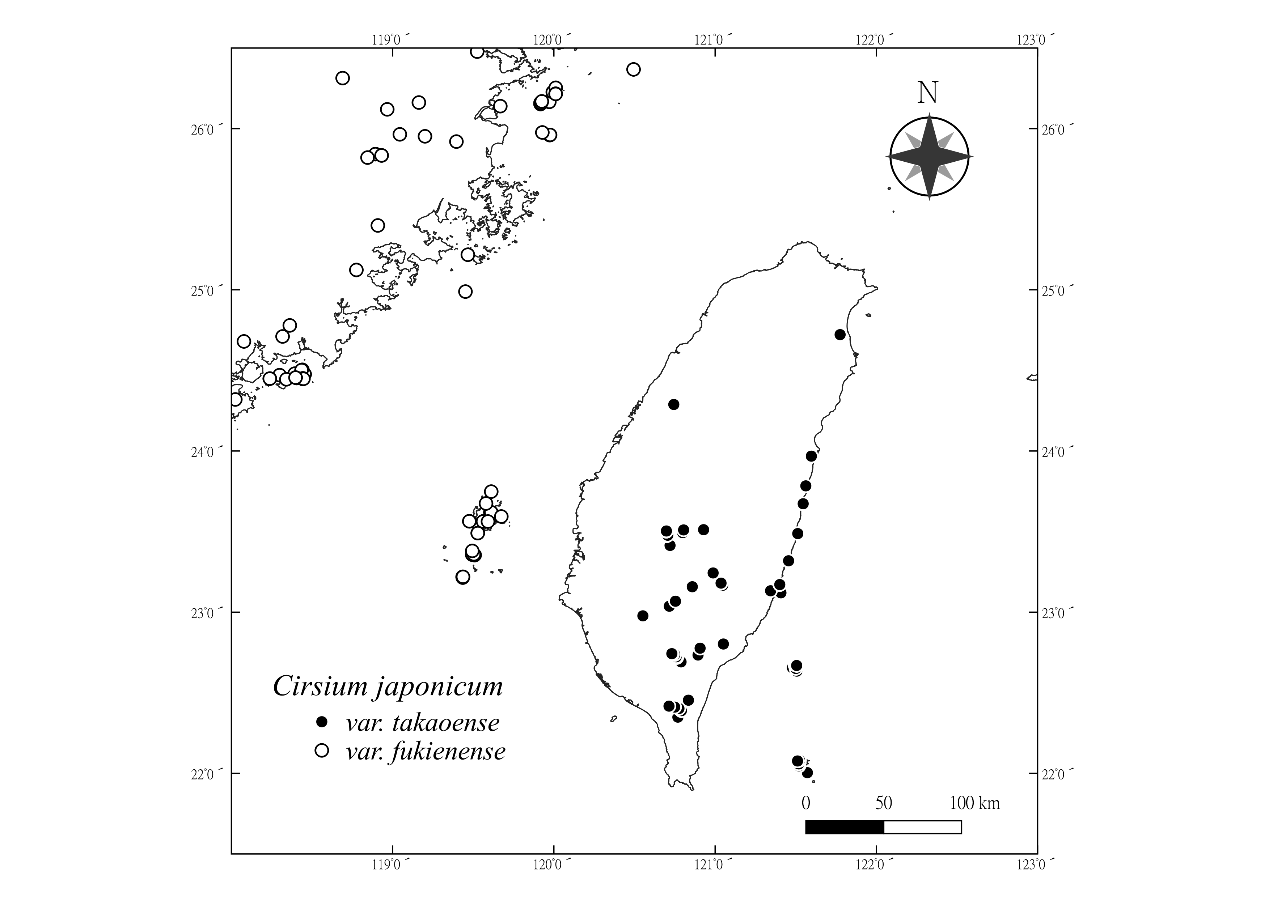


Supplementary Figure S4. Sampling localities for species distribution modeling (SDM).


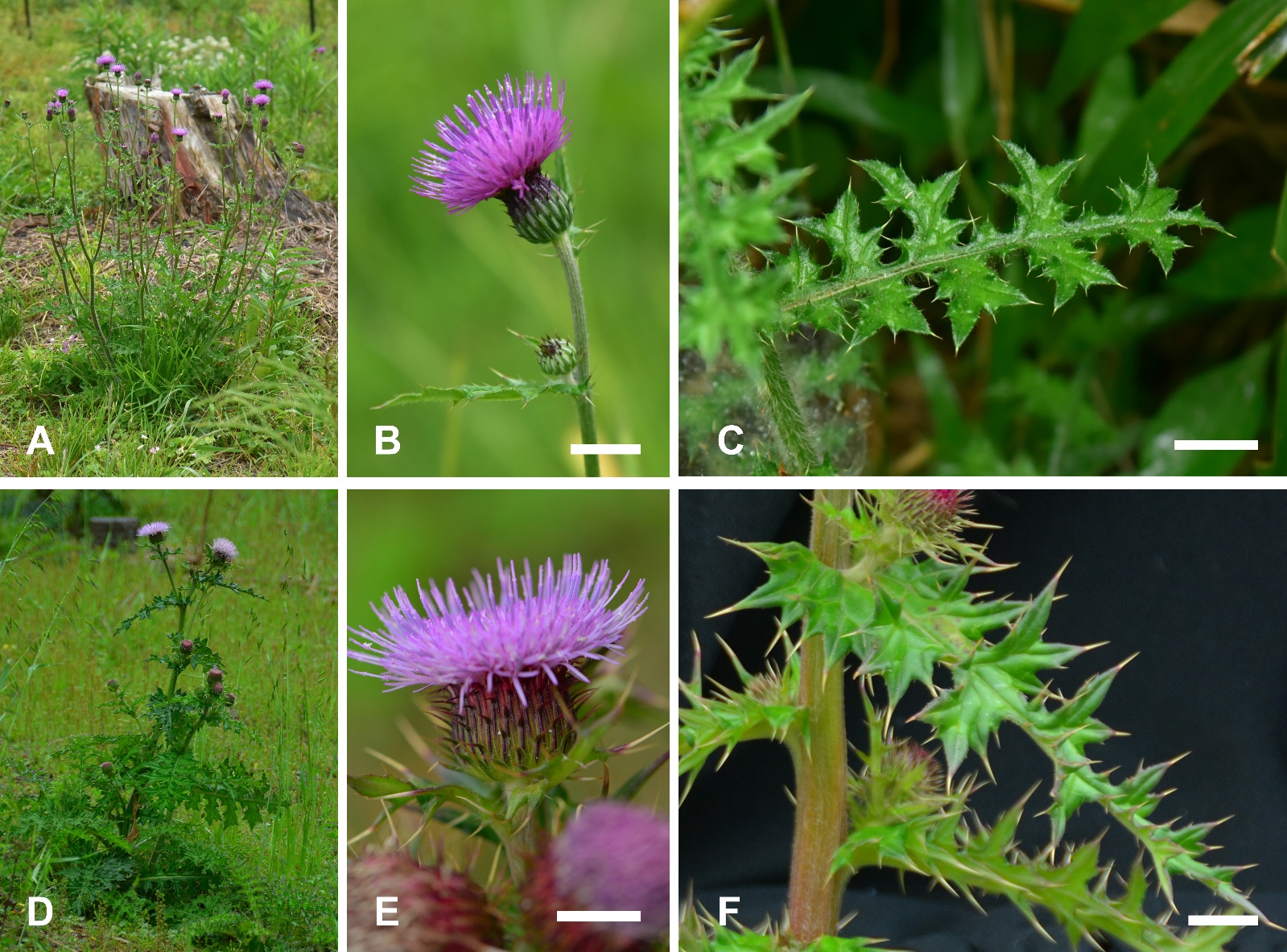


Supplementary Figure S5. Comparative morphology of *Cirsium japonicum* var. *japonicum* (A–C) and var. *fukienense* (D–F). (A, D) Habit. (B, E) Capitulum and upper stem. (C, F) Leaf lobes and marginal spines. Scale bars = 1 cm.


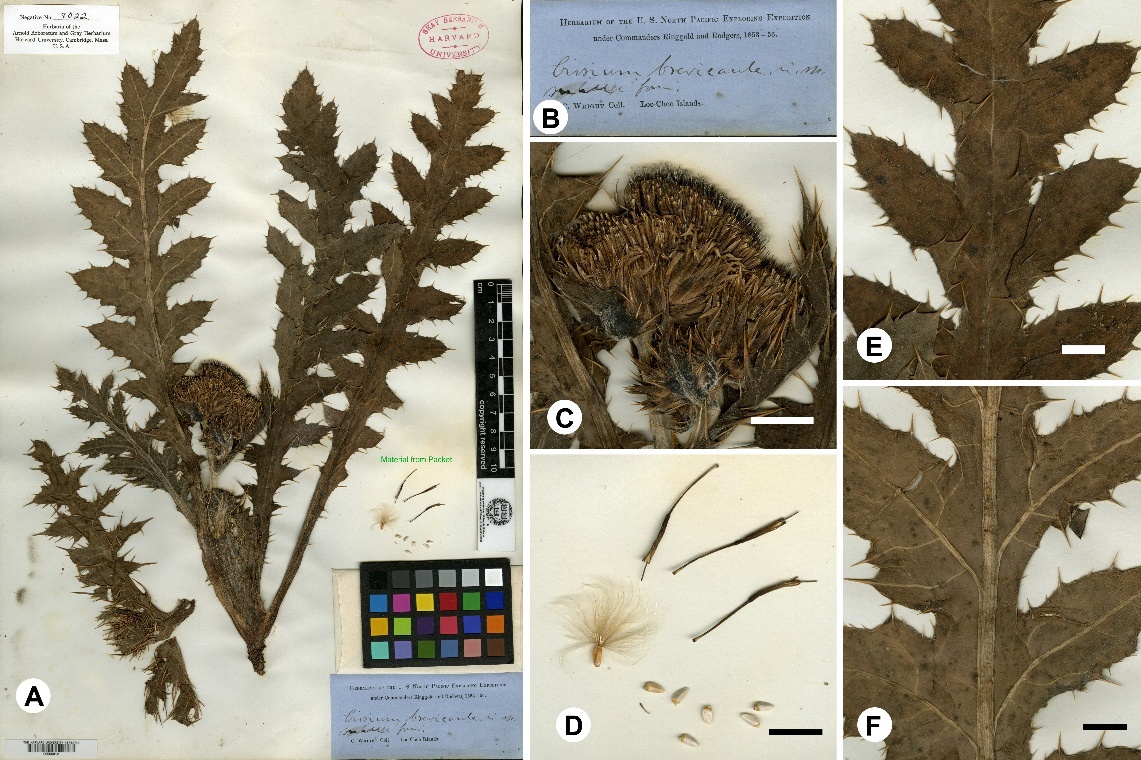


Supplementary Figure S6. *Cirsium brevicaule* A. Gray. Lectotype: HUH 6012!, designated here. (A) Whole specimen; (B) label; (C) capitulum; (D) reproductive structures; (E) adaxial leaf surface; (F) abaxial leaf surface. Scale bars: C–F = 1 cm.


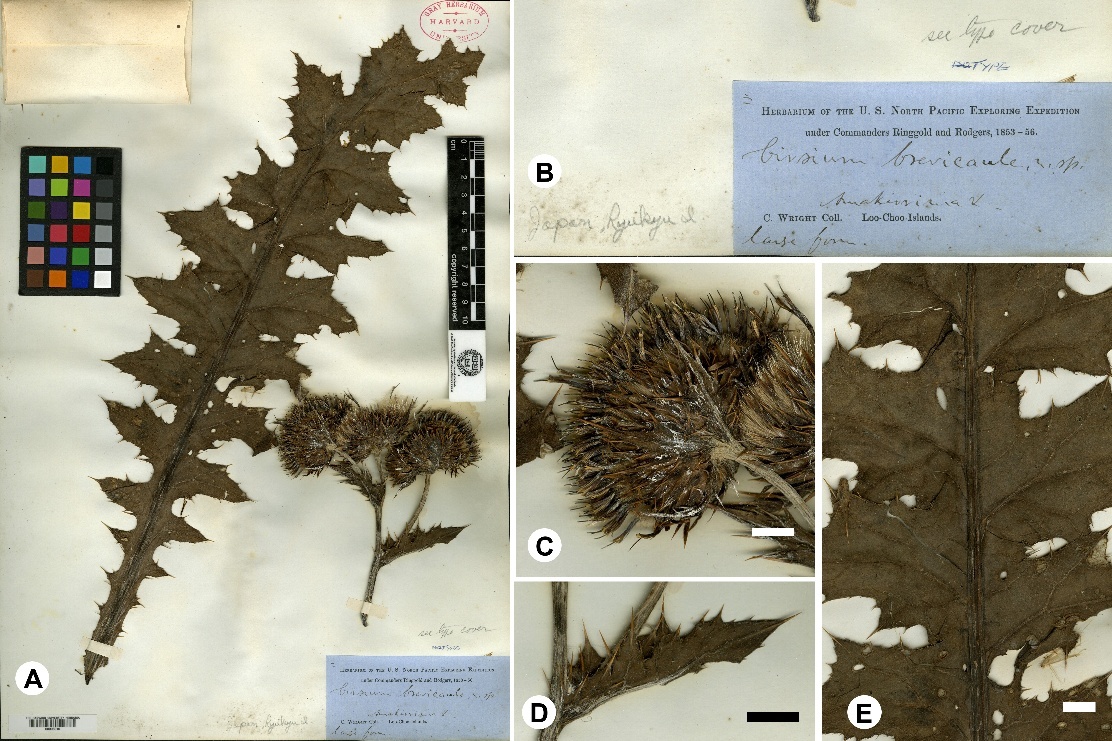


Supplementary Figure S7. *Cirsium brevicaule* A. Gray. Isolectotype: HUH 6010!. (A) Whole specimen; (B) label; (C) capitulum; (D, E) leaves. Scale bars: C–F = 1 cm.


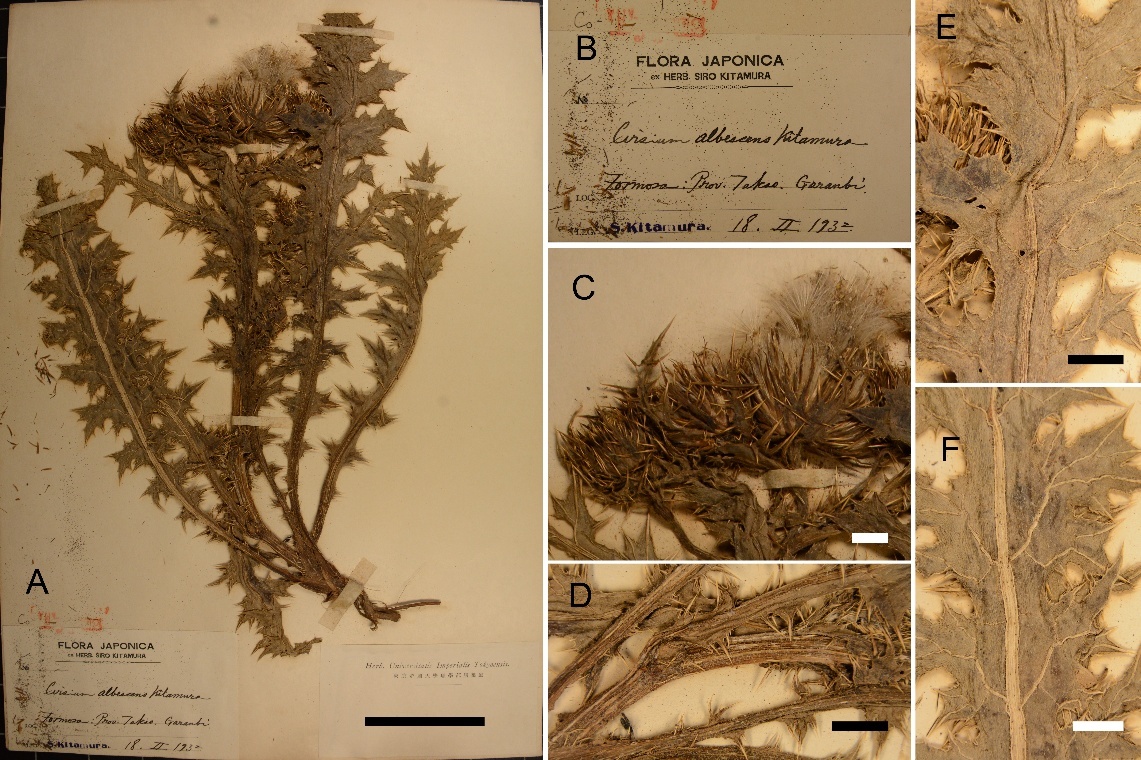


Supplementary Figure S8. *Cirsium japonicum* DC. var. *albescens* (Kitam.) Y. H. Tseng, P. C. Liao & Chih Y. Chang, comb. nov. Lectotype: TI!, designated here. (A) Whole specimen; (B) label; (C) capitulum; (D) stems; (E) adaxial leaf surface; (F) abaxial leaf surface. Scale bars: A = 5 cm; C–F = 1 cm.


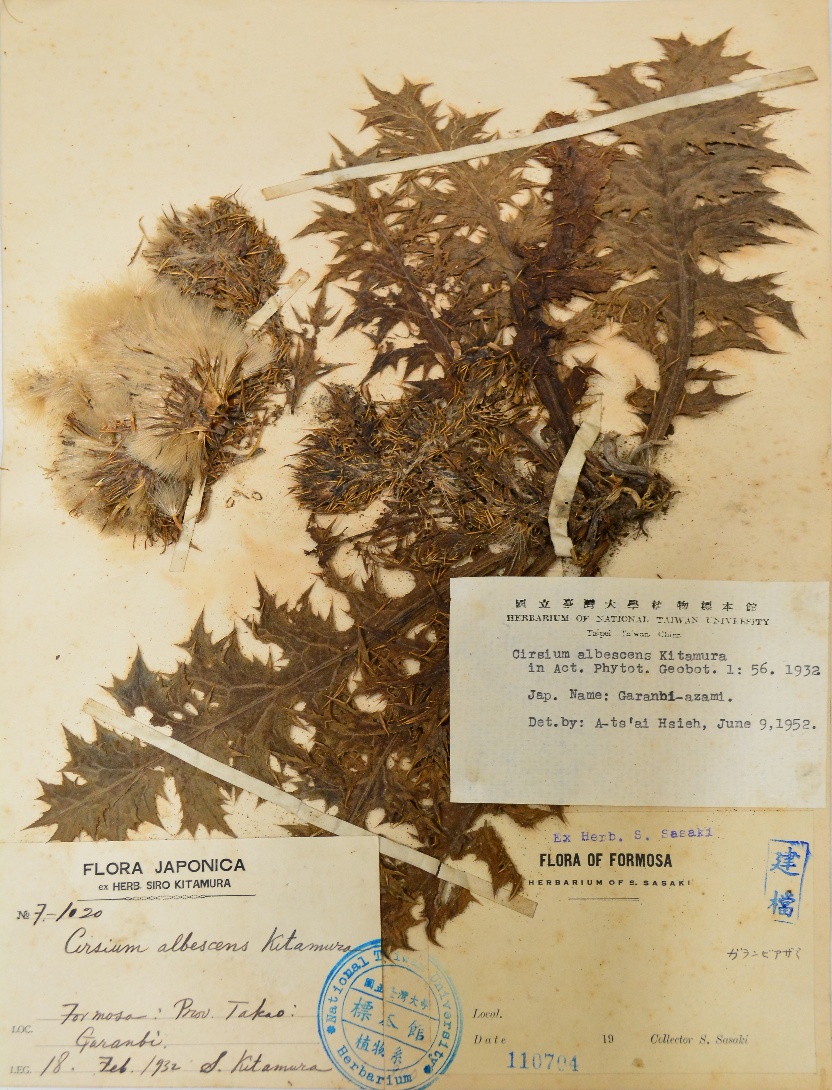


Supplementary Figure S9. *Cirsium japonicum* DC. var. *albescens* (Kitam.) Y. H. Tseng, P. C. Liao & Chih Y. Chang, comb. nov. isolectotype: TAI 110704!,


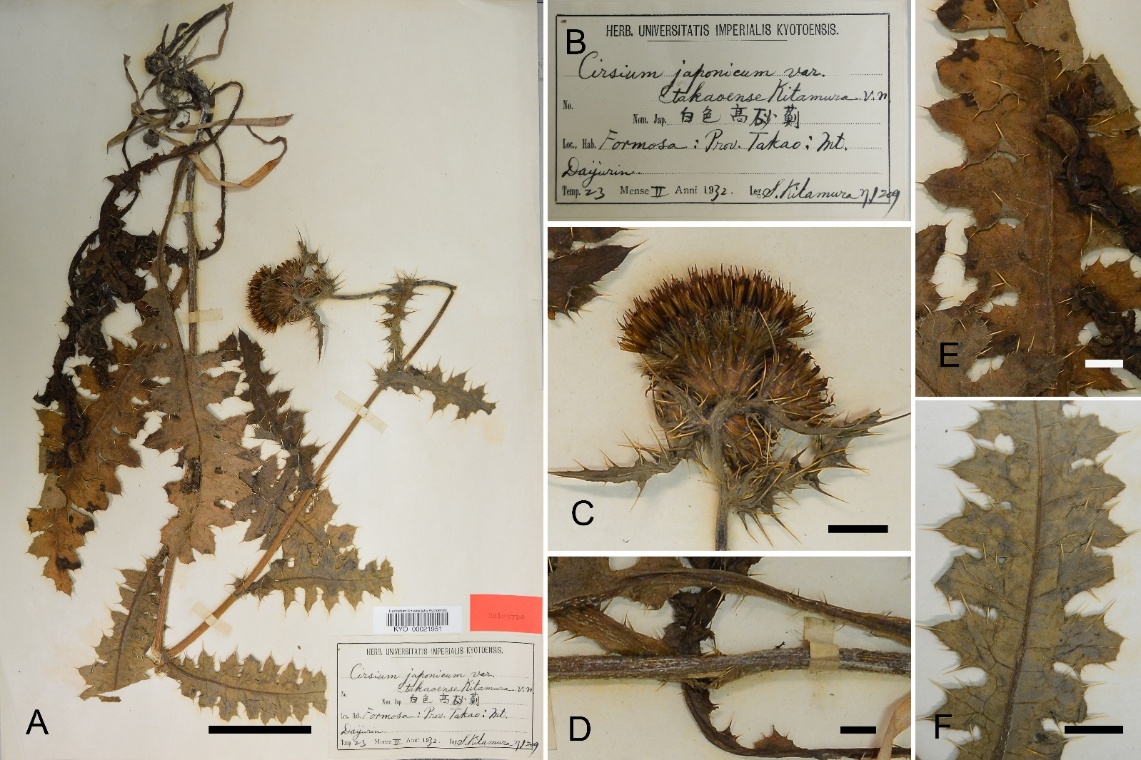


Supplementary Figure S10. *Cirsium japonicum* DC. var. *takaoense* Kitam. Lectotype: KYO 21951!, designated here. (A) Whole specimen; (B) label; (C) capitulum; (D) stem; (E) adaxial leaf surface; (F) abaxial leaf surface. Scale bars: A = 5 cm; C–F = 1 cm.


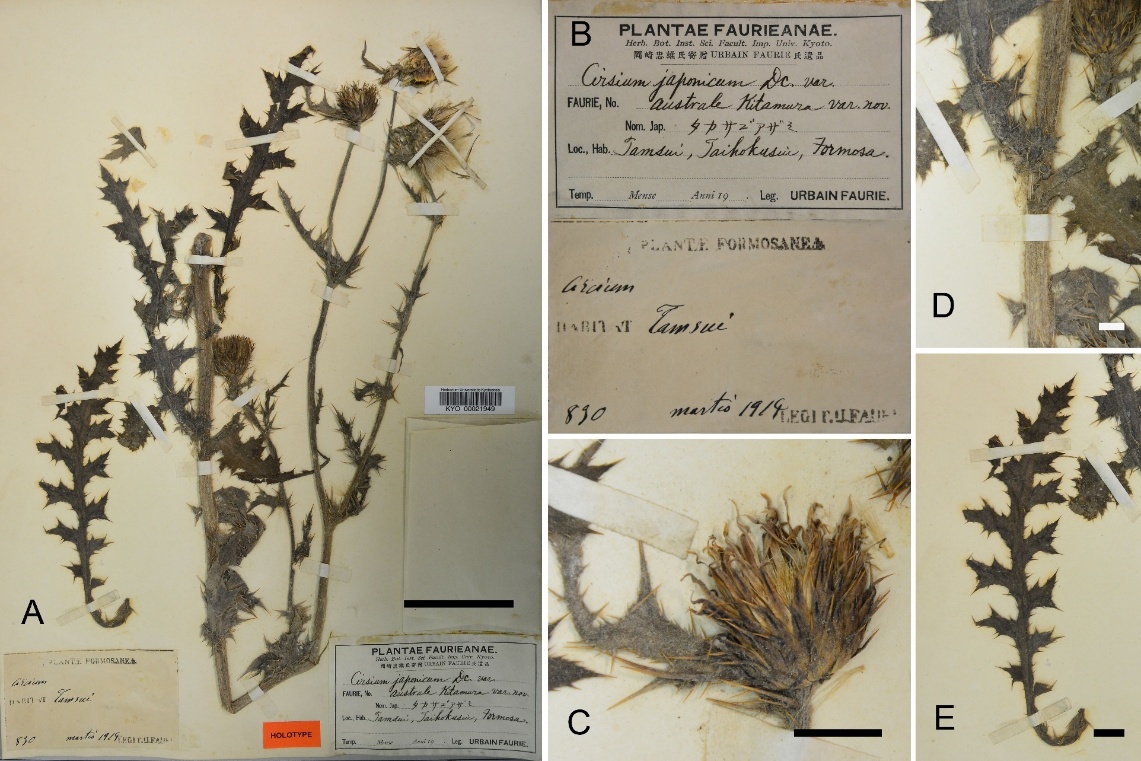


Supplementary Figure S11. *Cirsium japonicum* DC. var. *australe* Kitam. Lectotype: KYO 21951!, designated here. (A) Whole specimen; (B) label; (C) capitulum; (D) stem; (E) adaxial leaf surface; (F) abaxial leaf surface. Scale bars: A = 5 cm; C–F = 1 cm.


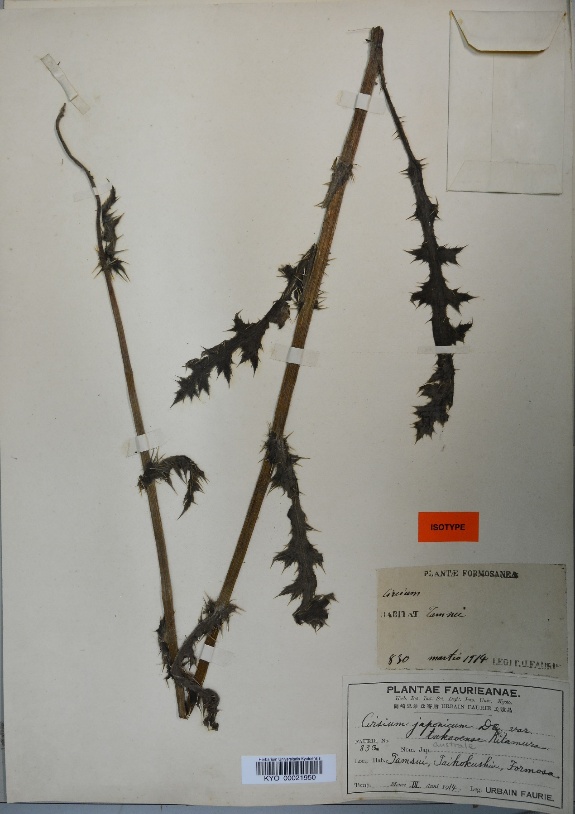


Supplementary Figure S12. *Cirsium japonicum* DC. var. *australe* Kitam. Isolectotype: KYO 21950!


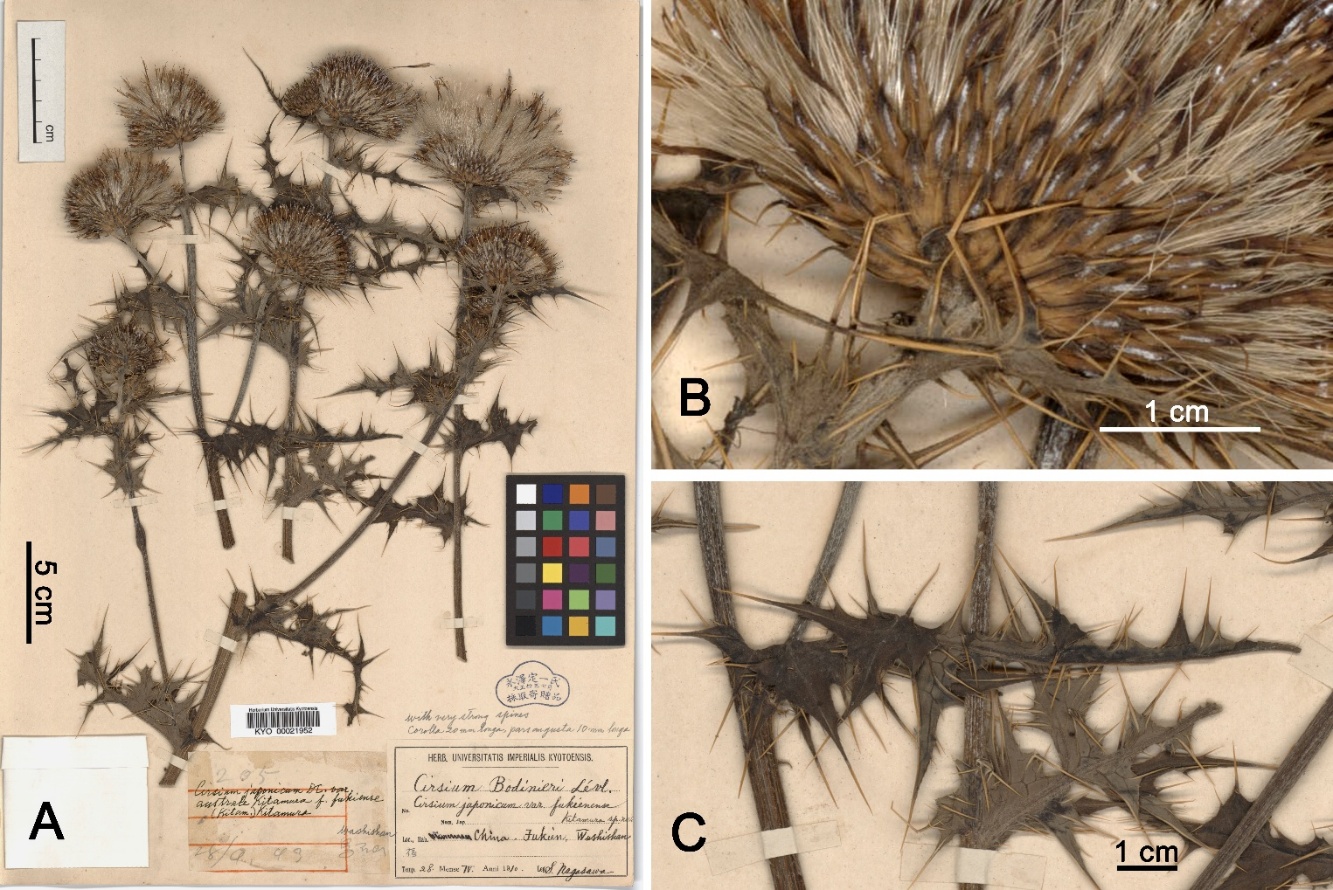


Supplementary Figure S13. *Cirsium japonicum* DC. var. *fukienense* Kitam. Lectotype: KYO 21952!, designated here. (A) Whole specimen; (B) capitulum; (C) leaf.

## Supplementary Table

Supplementary Table S1. Bioinformation and voucher of phylogenetic reconstruction.

| **Taxon** | **Raw data** | | **Trimmed** | | **Trinity (C/D, %)** | **cd-hit-est (C/D, %)** | **Collecting location** | **Code** | **Coordinate** | **Altitude (m)** | **Voucher** | **Herbarium** |
| --- | --- | --- | --- | --- | --- | --- | --- | --- | --- | --- | --- | --- |
|  | **Number of reads** | **GC%** | **Read (M)** | **GC%** |  |  |  |  |  |  |  |  |
| *C. japonicum* var. *albescens* | 43,966,372 | 45 | 18.1 | 44 | 90.6/32.1 | 90.3/10.6 | TAIWAN. Pingtung County: Bitou | BT | 22°06'N, 120°53'E | 77 | *ccy3173* | TCF |
|  | 40,126,720 | 51 | 3.6 | 44 | 27.0/6.2 | 26.9/2.2 | TAIWAN. Pingtung County: Kengzihnei | KZ | 21°54'N, 120°51'E | 35 | *ccy3515* | TNM |
| *C. japonicum* var. *takaoense* | 49,885,086 | 48 | 12.2 | 45 | 25.2/5.5 | 25.2/1.8 | TAIWAN. Chiayi County: Fenchihu | FC | 23°30'N, 120°41'E | 1,364 | *ccy3559* | TNM |
|  | 50,967,870 | 46 | 20.2 | 45 | 86.6/34.5 | 86.3/13.3 | TAIWAN. Kaohsiung City: Tengji | TJ | 23°02'N, 120°42'E | 1,127 | *ccy3807* | TCF |
|  | 53,816,156 | 48 | 12.0 | 45 | 17.7/2.9 | 17.6/1.3 | TAIWAN. Taitung County: Nanheng | NH | 23°10'N, 121°02'E | 991 | *ccy3835* | TCF |
|  | 46,736,488 | 52 | 3.7 | 45 | 21.8/4.6 | 21.7/1.5 | TAIWAN. Pingtung County: Wutai | WY | 22°44'N, 120°44'E | 977 | *ccy3560* | TNM |
|  | 51,440,434 | 45 | 21.5 | 45 | 86.2/37.8 | 85.8/15.8 | TAIWAN. Hualien County: Fengbin | FB | 23°40'N, 121°32'E | 21 | *ccy3629* | TNM |
|  | 53,912,776 | 46 | 21.8 | 45 | 81.5/32.4 | 81.3/11.2 | TAIWAN. Taitung County: Ludao | LT | 22°40'N, 121°30'E | 73 | *ccy3839* | TCF |
| *C. japonicum* var. *fukienense* | 54,631,670 | 45 | 22.8 | 45 | 86.2/36.9 | 85.9/15.3 | TAIWAN. Penghu County: Houliao | PH | 23°40'N, 119°34'E | 11 | *ccy3344* | TNM |
|  | 44,262,876 | 47 | 13.7 | 45 | 81.2/31.3 | 80.9/9.8 | TAIWAN. Lienchiang County: Xiju | MT | 25°58'N, 119°55'E | 110 | *ccy3541* | TCF |
|  | 46,709,226 | 47 | 11.8 | 45 | 9.4/1.4 | 9.4/0.5 | TAIWAN. Kinmen County: Jinhu | KM | 24°27'N, 118°23'E | 75 | *ccy3904* | TCF |
|  | 51,074,106 | 46 | 21.2 | 45 | 86.3/37.1 | 86.0/14.4 | TAIWAN. Lienchiang County: Beigan | MT | 26°12'N, 120°00'E | 46 | *ccy2257* | TCF |
| *C. japonicum* var. *australe* | 38,066,984 | 54 | 2.4 | 45 | 24.5/3.9 | 24.3/1.5 | TAIWAN. Miaoli County: Tongxiao | TX | 24°26'N, 120°41'E | 81 | *ccy2771* | TNM |
|  | 46,622,020 | 46 | 18.4 | 45 | 86.4/28.9 | 86.1/7.2 | TAIWAN. New Taipei City: Linkou | LK | 25°07'N, 121°18'E | 30 | *ccy3721* | TCF |
|  | 68,513,998 | 47 | 27.5 | 46 | 85.0/28.8 | 84.4/8.6 | TAIWAN. Taipei City: Yangmingshan | YM | 25°11'N, 121°31'E | 837 | *ccy3571* | TNM |
| *C. japonicum* var. *japonicum* | 41,302,000 | 46 | 16.9 | 45 | 84.9/33.5 | 84.7/11.3 | JAPAN. Fukuoka Prefecture: Fukuoka Experimental Forest | FKK | 33°38'N, 130°31'E | 73 | *ccy4204* | TCF |
|  | 47,526,726 | 46 | 18.8 | 45 | 87.4/32.9 | 87.1/10.7 | JAPAN. Kumamoto Prefecture: Aso | ASO | 32°53'N, 131°03'E | 394 | *ccy4220* | TCF |
| *C. brevicaule* | 43,221,610 | 46 | 15.7 | 44 | 78.6/25.2 | 78.5/6.9 | JAPAN. Kagoshima Prefecture: Amami | AMM | 28°28'N, 129°43'E | 15 | *ccy4163* | TCF |
|  | 39,985,382 | 46 | 15.0 | 44 | 79.4/26.9 | 79.3/8.4 | JAPAN. Kagoshima Prefecture: Yoron | YRN | 27°02'N 128°27'E | 25 | *ccy4166* | TCF |
|  | 50,157,726 | 48 | 20.4 | 46 | 82.2/29.7 | 82.0/10.3 | JAPAN. Okinawa Prefecture: Kunigami | OKN | 26°52'N, 128°15'E | 15 | *ccy4295* | TCF |
| *C. irumtiense* | 58,618,080 | 46 | 22.4 | 45 | 89.6/35.9 | 89.3/13.5 | JAPAN. Okinawa Prefecture: Miyakojima | MMY | 24°50'N, 125°12'E | 14 | *ccy4078* | TCF |
|  | 55,332,926 | 48 | 15.4 | 45 | 63.9/15.8 | 63.8/4.6 | JAPAN. Okinawa Prefecture: Ishigaki | ISG | 24°27'N, 124°08'E | 16 | *ccy4111* | TNM |
|  | 49,886,182 | 48 | 19.8 | 45 | 87.9/38.4 | 87.6/14.9 | JAPAN. Okinawa Prefecture: Yonaguni | YGN | 24°28'N, 123°00'E | 20 | *ccy4296* | TCF |
| *C. morii* | 54,837,686 | 45 | 23.0 | 45 | 85.1/39.3 | 84.8/15.9 | TAIWAN. Hualien County: Tianchang | TC | 24°01'N, 121°22'E | 1,303 | *ccy3360* | TCF |
|  | 71,123,946 | 48 | 29.3 | 45 | 89.0/36.4 | 87.1/9.7 | TAIWAN. Hualien County: Heping | TRK | 24°18'N, 121°44'E | 35 | *ccy4365* | TCF |
| *C. lineare* | 62,493,916 | 45 | 25.7 | 45 | 82.8/37.1 | 82.5/13.2 | TAIWAN. Miaoli County: Xihu | XH | 24°29'N, 120°45'E | 173 | *ccy3446* SRR30617342 | TNM |
|  | 37,079,400 | 47 | 10.9 | 45 | 44.3/15.1 | 44.0/3.3 | TAIWAN. Miaoli County: Tongxiao | TX | 24°26'N, 120°41'E | 81 | *ccy2770* SRR30617347 | TNM |
| *C. tatakaense* | 65,271,584 | 45 | 27.4 | 43 | 87.1/40.4 | 86.7/17.9 | TAIWAN. Nantou County: Luanantang | LAT | 23°42'N, 120°57'E | 2,476 | *ccy4022* SRR30617336 | TCF |
|  | 53,163,164 | 47 | 13.9 | 45 | 55.6/17.3 | 55.5/5.9 | TAIWAN. Chiayi County: Mt. Yushan | YS | 23°28'N, 120°55'E | 2,913 | *ccy3458* SRR30617340 | TCF |
| *C. kawakamii* | 57,811,986 | 47 | 17.1 | 45 | 68.6/31.0 | 68.4/6.5 | TAIWAN. Nantou County: Nenggao | NG | 24°02'N, 121°15'E | 2,751 | *ccy3861* SRR30617338 | TCF |
|  | 58,815,152 | 50 | 14.1 | 45 | 80.1/34.3 | 79.7/11.1 | TAIWAN. Nantou County: Mt. Xiaochilai | CL | 24°08'N, 121°17'E | 3,040 | *ccy3544* SRR30617339 | TNM |
| *C. pengii* | 57,811,986 | 46 | 26.7 | 45 | 91.1/38.7 | 90.9/17.0 | TAIWAN. New Taipei City: Mt. Lalashan | LLS | 24°43'N, 121°26'E | 1,736 | *ccy4054* SAMN43544268 | TCF |
|  | 53,346,610 | 45 | 22.4 | 44 | 90.2/45.9 | 89.7/21.1 | TAIWAN. New Taipei City: Mt. Lalashan | LLS | 24°43'N, 121°26'E | 1,736 | *ccy3995* SAMN43544266 | TCF |
| *Silybum marianum* | 32,523,260 | 44 | 29.7 | 44 | 85.8/45.7 | 85.4/21.2 | NCBI GenBank | -- | -- | -- | SRR12539244 | -- |
| *Cynara cardunculus* | 19,148,552 | 46 | 18.1 | 45 | 85.9/32.5 | 85.6/11.3 | NCBI GenBank | -- | -- | -- | SRR16295441 | -- |
| *Gerbera delavayi* | 28,597,092 | 45 | 21.8 | 44 | 91.9/50.1 | 91.9/24.1 | NCBI GenBank | -- | -- | -- | SRR5480948 | -- |
| *Nastanthus ventosus* | 26,354,302 | 46 | 21.5 | 45 | 83.1/44.1 | 82.7/20.3 | NCBI GenBank | -- | -- | -- | SRR12034794 | -- |

**Note.** C: Complete BUSCOs; D: Complete and duplicated BUSCOs.

Supplementary Table S2. Effective Sample Size (ESS) summary for divergence-time and Extended Bayesian skyline plot (EBSP) analyses.

| **Category** | **Representative parameters** | **Divergence-time analysis** | **EBSP – *C. japonicum* var. *takaonense*** | **EBSP – *C. japonicum* var. *fukienense*** |
| --- | --- | --- | --- | --- |
| **Tree model** | Posterior, likelihood, speciescoalescent, Tree.t | 792–19,330 | 347–71,052 | 1,316–72,250 |
| **Substitution models** | α-shape, relative rates (rateAC/rateAG/…); nucleotide frequencies; proportionInvariant | 912–19,817 | 90*–45,910 | 384–66,960 |
| **Clock model** | clockRate | 4,081–17,723 | 6,562–21,983 | 4,211–60,309 |
| **Priors** | *Divergence-time analysis:* Yule birth rate and two calibration priors. *EBSP:* Only the global prior term (“prior”). | 1,541–19,944 | 355 | 3,761 |
| **EBSP parameters** | Ne groups and skyline change parameters (populationMeans, popSizes) | N/A | 329–18,217 | 447–79,791 |

* Only 3 of the 114 substitution-model parameters had ESS < 200 (range 90–176). These correspond to low-information partitions and have negligible influence on posterior or demographic estimates. All key model parameters (posterior, clock rate, Ne, skyline groups) had ESS > 200.

Supplementary Table S3. Representative vouchers used to construct the reference tree for ancestral reconstruction of genome size in *Cirsium.*

| **Taxon** | **Voucher** | **Code** | **Herbarium** |
| --- | --- | --- | --- |
| *C. brevicaule* | *ccy4163* | AMM | TCF |
| *C. irumtiense* | *ccy4078* | MMY | TCF |
| *C. japonicum* var. *japonicum* | *ccy4204* | FKK | TCF |
| *C. japonicum* var. *australe* | *ccy3721* | LK | TCF |
| *C. japonicum* var. *fukienense* | *ccy3344* | PH | TNM |
| *C. japonicum* var. *takaoense* | *ccy3839* | LT | TCF |
| *C. japonicum* var. *albescens* | *ccy3173* | BT | TCF |

**Note.** Detailed information for each voucher is provided in Supplementary Table S1.

Supplementary Table S4. Genome size estimates (1C, 2C) and voucher information for the sampled *Cirsium* taxa.

| **Taxon** | **Collecting location** | **Coordinate** | **Altitude (m)** | **Voucher** | **Herbarium** | **CV** | **2C (pg)** | **2C (Mb)** | **1C (pg)** | **1C (Mb)** |
| --- | --- | --- | --- | --- | --- | --- | --- | --- | --- | --- |
| *C. japonicum* var. *japonicum* | JAPAN. Kumamoto Prefecture: Aso | 33°30'N, 131°3'E | 394 | *ccy4220* | TCF | 15.2 | 1.23 | 1,203 | 0.62 | 602 |
|  | JAPAN. Miyazaki Prefecture: Amanoiwato | 32°44'N, 131°21'E | 320 | *ccy4424-1* | TNM | 7.36 | 0.93 | 909 | 0.46 | 454 |
|  | JAPAN. Miyazaki Prefecture: Amanoiwato | 32°44'N, 131°21'E | 320 | *ccy4424-2* | TNM | 6.89 | 1.30 | 1273 | 0.65 | 637 |
|  | JAPAN. Miyazaki Prefecture: Amanoiwato | 32°44'N, 131°21'E | 320 | *ccy4424-3* | TNM | 6.93 | 1.33 | 1,301 | 0.67 | 651 |
| *C. japonicum* var. *albescens* | TAIWAN. Pingtung County: Eluanbi | 21°56'N, 120°51'E | 86 | *ccy4396-1* | TNM | 6.89 | 1.51 | 1,481 | 0.76 | 741 |
|  | TAIWAN. Pingtung County: Eluanbi | 21°56'N, 120°51'E | 86 | *ccy4396-2* | TNM | 6.83 | 1.79 | 1,747 | 0.89 | 873 |
|  | TAIWAN. Pingtung County: Eluanbi | 21°56'N, 120°51'E | 86 | *ccy4396-3* | TNM | 6.91 | 1.66 | 1,627 | 0.83 | 813 |
| *C. japonicum* var. *australe* | TAIWAN. Taipei City: Yangmingshan | 25°9'N, 121°33'E | 889 | *ccy4138* | TNM | 4.41 | 2.19 | 2,145 | 1.10 | 1,072 |
|  | TAIWAN. Miaoli County: Tongxiao | 24°32'N, 120°42'E | 149 | *ccy4399-1* | TNM | 6.98 | 1.38 | 1,347 | 0.69 | 674 |
|  | TAIWAN. Miaoli County: Tongxiao | 24°32'N, 120°42'E | 149 | *ccy4399-2* | TNM | 4.95 | 1.80 | 1,762 | 0.90 | 881 |
| *C. japonicum* var. *fukienense* | TAIWAN. Lienchiang County: Luobangshan | 26°13'N, 120°1'E | 46 | *ccy4389* | TNM | 4.69 | 2.31 | 2,262 | 1.16 | 1,131 |
|  | TAIWAN. Kinmen County: Jinhu | 24°26'N, 118°28'E | 30 | *ccy4397-1* | TNM | 6.87 | 1.82 | 1,775 | 0.91 | 888 |
|  | TAIWAN. Kinmen County: Jinhu | 24°26'N, 118°28'E | 30 | *ccy4397-2* | TNM | 6.8 | 1.61 | 1,573 | 0.80 | 786 |
|  | TAIWAN. Penghu County: Mt. Kuibishan | 23°36'N, 119°40'E | 17 | *ccy4398* | TNM | 6.89 | 1.91 | 1,865 | 0.95 | 933 |
| *C. japonicum* var. *takaoense* | TAIWAN. Chiayi County: Alishan | 23°29'N, 120°44'E | 1,351 | *ccy4392* | TNM | 6.38 | 1.11 | 1,088 | 0.56 | 544 |
|  | TAIWAN. Pingtung County: Wutai | 22°44'N, 120°44'E | 1,049 | *ccy4419* | TNM | 7.84 | 1.14 | 1,113 | 0.57 | 557 |
|  | TAIWAN. Taitung County: Green Island | 22°40'N, 121°30'E | 65 | *ccy4436-1* | TNM | 6.9 | 1.16 | 1,135 | 0.58 | 567 |
|  | TAIWAN. Taitung County: Green Island | 22°40'N, 121°30'E | 65 | *ccy4436-2* | TNM | 7.19 | 0.81 | 788 | 0.40 | 394 |
|  | TAIWAN. Taitung County: Green Island | 22°40'N, 121°30'E | 65 | *ccy4436-2* | TNM | 7.46 | 0.81 | 796 | 0.41 | 398 |
| *C. irumtiense* | JAPAN. Okinawa Prefecture: Ishigaki Island | 24°28'N, 124°9'E | 16 | *ccy4111-1* | TNM | 7.51 | 0.60 | 585 | 0.30 | 292 |
|  | JAPAN. Okinawa Prefecture: Ishigaki Island | 24°28'N, 124°9'E | 16 | *ccy4111-2* | TNM | 9.33 | 0.71 | 694 | 0.35 | 347 |
|  | JAPAN. Okinawa Prefecture: Ishigaki Island | 24°28'N, 124°9'E | 16 | *ccy4111-3* | TNM | 9.85 | 0.76 | 742 | 0.38 | 371 |
| *C. brevicaule* | JAPAN. Kagoshima Prefecture: Amami Ōshima | 28°28'N, 129°43'E | 15 | *ccy4164-1* | TNM | 13.4 | 1.87 | 1,828 | 0.93 | 914 |
|  | JAPAN. Kagoshima Prefecture: Amami Ōshima | 28°28'N, 129°43'E | 15 | *ccy4164-2* | TNM | 7.62 | 1.77 | 1,730 | 0.88 | 865 |
|  | JAPAN. Kagoshima Prefecture: Amami Ōshima | 28°28'N, 129°43'E | 15 | *ccy4164-3* | TNM | 6.52 | 2.16 | 2,113 | 1.08 | 1,057 |

**Note.** Genome-size values were calculated from propidium-iodide fluorescence ratios using the internal reference standard *Solanum lycopersicum* (2C = 2.0 pg ≈ 1.96 Gb [88, 89], assuming 1 pg = 978 Mb).

Supplementary Table S5. Summary of model performance and variable importance for two *Cirsium japonicum* varieties based on Maxent modeling.

| Taxon | Regularized  training gain | Iterations | Training  AUC | Top variable  (contribution %) | Top variable (permutation importance %) | Maximum training sensitivity plus specificity cumulative threshold |
| --- | --- | --- | --- | --- | --- | --- |
| var. *fukienense* | 2.0758 | 700 | 0.9383 | Precipitation of warmest quarter, BIO18 (30.83%) | Precipitation of warmest quarter, BIO18 (77.13%) | 29.4277 |
| var. *takaoense* | 1.7755 | 840 | 0.9403 | Mean diurnal range, BIO2 (48.10%) | Mean diurnal range, BIO2 (61.70%) | 20.8678 |

Supplementary Table S6. The examined specimens.

| 1. **Cirsium brevicaule** A.Gray, Mem. Amer. Acad. Arts. Sci. 6: 396. 1859. |
| --- |
| JAPAN. **Kagoshima Prefecture**: Ankyaba Observation Deck, *C.Y.Chang 4158* (TNM); Cape Ayamaru, *C.Y.Chang 4164* (TNM); China-chō, *J.Haginiwa JH011150* (TNS). **Okinawa Prefecture**: Hedo Cape, *C.Y.Chang 4294* (TNM); Manzamo, *C.Y.Chang 4145* (TNM); Kunigami-son, *H.Ikeda 93032051* (TNS); same loc., *Y.Kadota 61011* (TNS); Shuri-shi, *T.Ito 799* (TNS); same loc., *S.Sacaguchi 108* (TNS); Nago-shi, *H.Koyama 536* (TNS); Tokunoshima Island, *J.Haginiwa JH011149* (TNS); Iheyajima Island, *G.Kokubugata 10705* (TNS). |
| 2. **Cirsium irumtiense** Kitam., Acta Phytotax. Geobot. 2(1): 41. 1933. |
| JAPAN. **Okinawa Prefecture**: Miyako Island, Muiger, *C.Y.Chang 4092* (TNM); Irabu Island, Daishu Shrine, *C.Y.Chang 4075* (TNM); Ishigaki Island, *C.Y.Chang 4070* (TNM); same loc., *J.Haginiwa JH011155* (TNS); Kabira Bay, *C.Y.Chang 4111* (TNM); Ishigaki-jima, Ishigaki City, *J.Haginiwa JH011153* (TNS); Hateruma-jima, Yaeyama-gun, Taketomi Town, *J.Haginiwa JH030504* (TNS). |
| 3. **Cirsium japonicum** DC., Prodr. [A. P. de Candolle] 6: 640. 1838. |
| 3a. var. **albescens** (Kitam.) Y.H.Tseng, P.C.Liao & Chih Y.Chang, *comb. nov.* |
| TAIWAN. **Pingtung County**: Garanbi, Kasluin, *T.Kawakami s.n.* (TAIF); same loc., *S.Kitamura F-1020* (TAI); same loc., *Y.B.Zheng s.n.* (TAIF); Jiupeng Village, *C.I Peng 7830* (HAST); Fengchuisha, *C.I Peng 5516* (HAST); same loc., *T.C.Chen 4557* (TAIF); same loc., *C.Y.Chang 398* (TNM); Eluanbi, *C.Y.Chang 4396* (TCF); Longpan Scenic Area, *C.I Peng 16248* (HAST); same loc., *C.Y.Chang 180* (TNM); Xiaolongpan Park, *C.Y.Chang 878* (TCF); Mt. Mudanchishan, *Y.C.Lin 1022* (TCF); Sheding Nature Park, *C.I Peng 16211* (HAST); same loc., *S.W.Chung 2338* (TAIF); same loc., *J.Z.Chen 7359* (TAIF); same loc., *C.Y.Chang 3090* (TNM); Nanren Lake, *S.H.Wu 1106* (HAST); Nanrenshan, *J.G.Liu 70* (TAIF); same loc., *H.C.Liang 1833* (HAST); same loc., *C.I Peng 7791* (HAST); Nanrenshan–Jiupeng, *C.I Peng 7817* (HAST); Baoli Farm–Mudan, *C.M.Wang 3087* (HAST); Gaoshi–Mudan, *C.M.Wang 3974* (TNM); Mt. Gaoshifo, *Y.C.Kao 692* (HAST); Mt. Dajianshi, *Y.Tateishi 21718* (TAI); Jialuo River, *C.I Peng 16603* (TAI); Gaoshifo, *C.C.Chen 539* (TAI); Gaoshi, *T.T.Chen et al. 3264* (TAIF); Dajianshan Trail, *K.C.Yang s.n.* (TAIF); Jiupeng–Wushuixi, *C.M.Wang 2034* (TNM); Kenting, *M.T.Kao s.n.* (TNM); Mt. Silinge, *C.M.Wang 12672* (TNM); same loc., *T.Y.Aleck Yang 21474* (TNM); same loc., *Y.S.Wu 49* (CHIA); same loc., *C.Y.Chang 1104* (TCF, TNM); Longzipu, *C.Y.Chang 180* (TCF, TNM); Syuhai Grassland, *C.Y.Chang 389*, *C.Y.Chang 462* (TCF); Provincial Road 26, *C.Y.Chang 410* (TNM); Mt. Santai, *C.M.Wang 13537* (TNM); Longkeng, *S.Z.Yang 26486* (PPI); Kengtzunei, *C.Y.Chang 3515*, *C.Y.Chang 3666* (TNM); Yungching, *C.Y.Chang 1691* (TNM); same loc., *C.Y.Chang 2833* (TCF); Cape Chufengpi, *C.Y.Chang 3126* (TNM); Pitou Grasslands, *C.Y.Chang 3173* (TNM); Nanjen Fishery Harbor, *C.Y.Chang 3300*, *C.Y.Chang 3434* (TNM); Hsuhai Prairie, *C.Y.Chang 2785* (TNM); Mt. Dasianshihshan road, Fanlian–Huanshan trail, *C.Y.Chang 3391* (TCF). |
| 3b. var. **takaoense** Kitam., Acta Phytotax. Geobot. 1(1): 57. 1932. |
| TAIWAN. **Taichung City**: Houli Pili Temple, *C.Y.Chang 870* (TNM). **Chiayi County**: Puyana Forest Road, *S.M.Liu 657* (HAST); Alishan, *S.Y.Lu 16025* (TAIF); Tefuye, *M.J.Chung x11701* (TAIF); Fenqihu, *C.H.Chen 8611* (TNM); same loc., *W.T.Cheng 1* (TCF); same loc., *C.Y.Chang 3559* (TNM); Shalixian Creek, *Huang 3316* (TNU); Lijia, *L.H.Li 83* (TAIE); Fenqihu Cultural History Museum, *C.Y.Chang 1085*, *C.Y.Chang 1086* (TNM); same loc., *C.Y.Chang 1083* (TCF); Teenyana Community, *C.Y.Chang 4392* (TNM). **Kaohsiung City**: Mt. Hsiaokuanshan Pond Shenchih, *C.Y.Chang 3339* (TNM); Tengzhi Forest Road 12 km, *C.Y.Chang 408*, *C.Y.Chang 4420* (TCF); Tengzhi Damen-Zhuwu Sales Area, *C.Y.Chang 985* (TNM); Riguang Forest, *C.Y.Chang 1111* (TNM). **Pingtung County**: Dahanshan Forest Road 15.2 km, *K.F.Chung 1296* (HAST); Dahanshan Forest Road 21 km, *C.I Peng 23191* (HAST); same loc., *C.Y.Chang 237* (TNM); Dahanshan Forest Road 24–25 km, *Q.Y.Li 1011* (TAIE); Xiaogui Lake, *S.Z.Yang 7241*, *S.Z.Yang 30017* (PPI); Xiaogui Lake Forest Road, *K.P.Hsieh 2371* (TAIF); Xiaogui Lake Forest Road 3 km, *C.Y.Chang 947* (TCF); Aluwan Ancient Trail, *C.Y.Chang 192* (TNM); Ali, *C.C.Lu s.n.* (TNU); same loc., *S.Z.Yang 10107* (PPI); same loc., *C.Y.Chang 239* (TCF); Jinshuiying, *S.Mori 3143* (TAIF); same loc., *C.Y.Yeh 2083* (TAIE); same loc., *C.F.Chen 354* (PPI); Wutai, *H.L.Chiang 1940* (TAIF); Wutai–Ali, *W.P.Leu 998* (HAST); same loc., *C.I Peng 10195*, *C.I Peng 10186* (HAST); same loc., *C.Y.Chang 946* (TCF); same loc., *C.Y.Chang 3560* (TNM); Tianzhuling, *C.Y.Chang 4419* (TCF). **Hualien County**: Ji’an River Estuary, *S.H.Chen s.n.* (TAIF); Shuilian, *C.M.Wang 5736* (TNM); Shitiping, *C.S.Wu 6-04* (TNM); Chinpuchihtzu Historic Trail, *C.Y.Chang 3629* (TNM). **Taitung County**: Sanxiantai, *C.Y.Chang 1176* (TCF); same loc., *C.Y.Chang 1636*, *C.Y.Chang 2728* (TNM); Dahanshan Forest Road, *K.W.Li 185* (PPI); Xiaotianchi, *H.L.Chiang s.n.* (TAIF); Xiaogui Lake Forest Road, *K.P.Hsieh 2371* (PPI); Xiangyang, *C.K.Chen 877* (TCF); same loc., *C.Y.Chang 876* (TCF); Lijia Forest Road 21 km, *J.C.Kao 186* (HAST); Lidao, *C.Y.Chang 1163* (TCF, TNM); Changbin Township, *K.P.Hsieh 1255* (PPI); Qingqing Grassland, *M.J.Chung 2545* (TAIF); Youzihu, *C.M.Wang 7182* (TNM); same loc., *C.Y.Chang 4436* (TNM); Mt. Xiangaishan, *M.J.Chung 4786* (TAIF); Mt. Hongtoushan Trail, *C.Y.Chang 514* (TNM); Langdao Roadside, *C.E.Chang 14768* (PPI); Jinshuiying, *K.P.Hsieh 931* (PPI); Mt. Chachayalaishan, *C.F.Chen 1684* (PPI); Xinhua–Tusakan, *S.W.Chung 6230* (TAIF); Green Island Academia Sinica Trail 200 m, *C.S.Wu 2676* (TNM); Green Island Fanche Nose, *C.S.Wu 2679* (TNM); Green Island Nanliao, *K.P.Hsieh 1836* (PPI); Green Island Wankou Road, *H.P.Su s.n.* (TAIF); Green Island Grassland, *C.C.Chen 36* (PPI); Provincial Highway 20 E Section, *M.J.Chung w12902* (TNM); Mt. Wutoushan, *C.Hsien 437* (PPI); Mt. Mantoushan, *M.J.Chung 4047* (TAIF); Orchid Island Xiaotianchi, *W.P.Leu 1367* (HAST); Orchid Island Tank Rock, *Z.Y.Yang 11898* (TNM); Orchid Island Hongtou–Tianchi, *C.I Peng 5225* (HAST); Orchid Island Langdao, *C.I Peng 7643* (HAST); Orchid Island, *C.E.Chang 20335* (PPI); same loc., *F.Y.Lu 2270* (CHIA); Rainbow Waterfall, *C.Y.Chang 2803* (TNM); Hungtou Village, *C.Y.Chang 516* (TNM); Da-Tong Vacation Hotel, *C.Y.Chang 2861* (TCF); Niutoushan, *C.Y.Chang 3839* (TNM). |
| CHINA. **Jiangsu Province**: Shiziling, *G.F.Ruan s.n.* (PE); Chongshan, northern foot, *W.X.Wu 5639* (NAS); Ming Xiaoling Mausoleum, *G.Yao 8107* (HHBG). **Guangxi Zhuang Autonomous Region**: *Z.Z.Chen 51422* (IBSC); Jiuwan Mountains, Sanfang, *Beijing Expedition 892087* (PE); Jiuwan Dashan, *D.Z.Chen 161* (KUN). **Sichuan Province**: *C.W.Yao 3976* (PE). |
| 3c. var. **australe** Kitam., *Cirs*. Nov. Orient.-Asiat. 12. 1931. |
| TAIWAN. **Keelung City**: Keelung, *T.Kawakami s.n.* (TAIF); Velung, *B.Hayata s.n.* (TAIF); Patoutze, *C.I Peng 7576* (HAST); Patoutzu Wangyuku Trail, *C.Y.Chang 2767* (TNM). **Taipei City**: Yangmingshan, *J.L.Panero 6414* (HAST); Mt. Tatunshan, *T.Y.Liu 213* (HAST); Yangmingshan National Park, *W.P.Leu 510* (HAST); Dajianchi, *Y.Y.Huang 288* (HAST); Xidi, *S.Sasaki s.n.* (TAIF); Mt. Qixingshan, *C.W.Tsai s-2* (TAIF); same loc., *W.P.Ho s.n.* (TNU); Tamsui, *S.Y.Lu 15346* (TAIF); Yangmingshan, alt. 700–1,000 m, *W.B.Lu s.n.* (TNM); Ferry Bow, *Z.K.He s.n.* (TNU); Yangjin Road, *Huang 1186* (TNU); Menghuan Lake to Qixing Park, *C.Y.Chang 4138* (TNM). **New Taipei City**: Bat Cave, *C.I Peng 22360* (HAST); Shiliufenzi, *C.I Peng 6782* (HAST); Sandiaojiao Lighthouse, *H.C.Liang 2749* (HAST); Mt. Lingjiaoshan, *H.L.Chiang 41* (TAIF); Shimen, *W.F.Ho 777* (TAIF); 2nd Nuclear Power Plant, *M.J.Chung 5473* (TAIF); Fugueijiao, *M.J.Chung 3800* (TAIF); Fukueichiao Lighthouse, *C.Y.Chang 2782* (TCF, TNM); Mucha, *S.P.Li 75* (TNM); Nangang, *R.F.Wang s.n.* (TNU); Jinshan Tiaoshi Coast, *C.H.Chen 5289* (TAIE); same loc., *C.Y.Chang 4121* (TNM); Bitoujiao Park, *C.Y.Chang 1343* (TCF); Yeliu, *Y.T.Lin s.n.* (TNU); Shuiniukeng, *C.Y.Chang 1486*, *C.Y.Chang 2407* (TNM); Binhai Highway Parking Lot 2, *C.Y.Chang 4124* (TNM). **Hsinchu County**: Shanchiao, *C.I Peng 10384* (HAST); Lianhua Temple, *C.I Peng 11515* (HAST); same loc., *L.Y.Hung LYH203* (TNM); Xinfeng, *H.L.Chiang 1630* (TAIF). **Miaoli County**: Ziyun Temple, *C.H.Lin 158* (HAST); County Highway 128, *C.I Peng 13957* (HAST); Lingding, *C.M.Wang 14349* (TNM); same loc., *C.Y.Chang 428*, *C.Y.Chang 429*, *C.Y.Chang 435*, *C.Y.Chang 470*, *C.Y.Chang 473*, *C.Y.Chang 1124* (TCF, TNM), *C.Y.Chang 1650*, *C.Y.Chang 4134* (TNM), *C.Y.Chang 4399* (TCF); Tongxiao Town Thirteen Cemetery, *Z.W.Hsu 8191* (TAIE); Tongxiao No. 14 Cemetery, *C.Y.Chang 2771* (TNM); same loc., *C.Y.Chang 2772* (TCF); Houlong Bantianliao, *C.D.Yang 680* (TAIE); Miaoli City The Second Cemetery, *C.Y.Chang 1722* (TNM). **Taichung City**: Tiezhenshan, *M.J.Chung 3853* (TAIF); Nanshikeng, *C.M.Wang 13598* (TNM). **Changhua County**: Mt. Baguashan, *Y.Shimada 6180* (HAST); Changhua, *C.Chang s.n.* (TCF). **Nantou County**: Yunhai–Tianchi, *C.C.Liao 1347* (HAST); Shenmu Village 31 Forest Class, *C.I Peng 6587* (HAST); Wushe–Liying, *S.Saito s.n.* (TI); Wushe–Qingjing Farm, *T.Namba 2641* (TI); Danda Forest Road 4–6 km, *C.M.Wang 9765* (TNM); Danda Forest Road, *T.Y.Aleck Yang 6036* (TNM). |
| CHINA. **Zhejiang Province**: Hangzhou, *s.n. 95* (PE). **Hubei Province**: Luojia Hill, *C.H.Qian 1353* (IBSC). **Fujian Province**: Jiangshi Village, *X.L.Hou 91674* (AU); Longxishan Nature Reserve, *Longxishan Expedition 633* (PE); Changting Beishan, *Y.Ling 5689* (PE). **Guangdong Province**: Conghua County, Sanjiaoshan, *W.T.Tsang 25114* (IBSC); North River Region, *E.D.Merrill 2330* (PE); Xiaobai Mountain, *L.Deng 6184* (PE). **Anhui Province**: Huangshan, Santongting Pavilion, *East China Station Team 6447* (PE). |
| 3d. var. **fukienense** Kitam., Acta Phytotax. Geobot. 1(2): 149. 1932. |
| TAIWAN. **Penghu County**: Penghu, *J.C.Lin 272* (TAIF); same loc., *M.F.Lou 6* (TAIF); same loc., *C.T.Chen 332* (PPI); Magong City, beach, *C.Y.Chang 542* (TNM); Table Island, *C.Y.Chang 3274* (TNM); Jingueitou Fort, *C.Y.Chang 1821* (TNM); Xiyu Township, Shitai bait cannon, *C.Y.Chang 1841* (TNM); Wangan Township, Dongan, *T.Y.Aleck Yang 20454* (TNM); Guibikanwei, *C.Y.Chang 3348* (TNM); Tutikungkang Harbour, *C.Y.Chang 3351*, *C.Y.Chang 3352* (TNM); Qimei Township, Qimei Airport, *M.J.Jung 3262* (TAIF); Northwest Gulf, *C.Y.Chang 1832* (TNM); Huxi Township, Shagang, *K.C.Yang 4838* (TAIF); Kueipishan, *C.H.Chen 727* (HAST) ; same loc., *C.Y.Chang 4398* (TCF). **Kinmen County**: Jinsha Township, Pishan, *K.C.Lu 5688* (TCF); Pubian, *S.T.Chiu 5107* (TNM, PPI); Shanhou, *S.T.Chiu 6620* (TNM); Tianpu Reservoir, *C.M.Wang 5696* (TNM); Mt. Wuhushan, *W.L.Hsieh s.n.* (CHIA); Jinhu Township, Hulung, *C.M.Wang 2547* (HAST, TAIF, TNM); Mt. Nantaiwushan, *S.C.Liu 1058* (TNU); Jinning Township, Tzuhu, *T.T.Chen 8124* (TAIF); Agricultural Research Institute, *C.M.Wang 1881* (TNM); Lieyu Township, Hongtugou, *C.Y.Chang 728* (TCF, TNM); Fengshang Xunjian-si, *C.Y.Chang 4397* (TCF); Yangtsu, *F.Y.Lu H2657* (CHIA). **Lienchiang County**: Nangan Township, Huangkuanyu Island, *W.H.Wu et al. 297* (TAIF); Mazu Village, *T.Y.Liu 543* (HAST); same loc., *C.Y.Chang 2249* (TNM); Shengtian Park, *C.M.Wang 10010* (TNM); Furen Village, Guanhai Road, *C.Y.Chang 2240* (TNM); Beigan Township, *Y.P.Cheng 3743* (TAIF); same loc., *H.L.Chiang 2125* (TAIF); Jinyu Island, *C.H.Chen 10028* (TNM); Daqiu Island, *C.M.Wang 10081* (TNM); Mt. Luoshan Trail, *C.Y.Chang 2257* (TNM); same loc., *C.Y.Chang 4389* (TCF); Chukuang Township, Taping to lighthouse, *T.Y.Liu 666* (HAST); Dongyin Township, Dongyin Island, *P.F.Lu 6187* (TAIF). |
| CHINA. **Hong Kong SAR**: Tap Mun Island, *S.Y.Hu 11945* (PE); Kadoorie Farm & Botanic Garden, *C.Y.Chang 4421* (TNM). **Fujian Province**: Xiamen City, Nanputuo Temple, *C.K.Tseng 688* (AU); Leshan, *Z.G.Dong 60* (IBSC); Xiamen University Xiang’an Campus, *Y.Shen 60072* (AU); Jinguang Lake, *X.Y.Li 130429002* (AU). |
